# A Potent and Selective Dual Inhibitor of AXL and MERTK Possesses Both Immunomodulatory and Tumor-Targeted Activity

**DOI:** 10.3389/fonc.2020.598477

**Published:** 2020-12-07

**Authors:** Jonathan Rios-Doria, Margaret Favata, Kerri Lasky, Patricia Feldman, Yvonne Lo, Gengjie Yang, Christina Stevens, Xiaoming Wen, Sarita Sehra, Kamna Katiyar, Ke Liu, Richard Wynn, Jennifer J. Harris, Min Ye, Susan Spitz, Xiaozhao Wang, Chunhong He, Yun-Long Li, Wenqing Yao, Maryanne Covington, Peggy Scherle, Holly Koblish

**Affiliations:** Incyte Research Institute, Wilmington, DE, United States

**Keywords:** AXL, MERTK, PD-L1, immunotherapy, dual inhibitor, antitumor activity, small molecule

## Abstract

TYRO3, AXL, and MERTK constitute the TAM family of receptor tyrosine kinases, which play important roles in tumor growth, survival, cell adhesion, as well as innate immunity, phagocytosis, and immune-suppressive activity. Therefore, targeting both AXL and MERTK kinases may directly impact tumor growth and relieve immunosuppression. We describe here the discovery of INCB081776, a potent and selective dual inhibitor of AXL and MERTK that is currently in phase 1 clinical trials. In cellular assays, INCB081776 effectively blocked autophosphorylation of AXL or MERTK with low nanomolar half maximal inhibitory concentration values in tumor cells and Ba/F3 cells transfected with constitutively active AXL or MERTK. INCB081776 inhibited activation of MERTK in primary human macrophages and partially reversed M2 macrophage–mediated suppression of T-cell proliferation, which was associated with increased interferon-γ production. *In vivo*, the antitumor activity of INCB081776 was enhanced in combination with checkpoint blockade in syngeneic models, and resulted in increased proliferation of intratumoral CD4^+^ and CD8^+^ T cells. Finally, antitumor activity of INCB081776 was observed in a subset of sarcoma patient–derived xenograft models, which was linked with inhibition of phospho-AKT. These data support the potential therapeutic utility of INCB081776 as an immunotherapeutic agent capable of both enhancing tumor immune surveillance and blocking tumor cell survival mechanisms.

## Introduction

The TAM kinases (TYRO3, AXL, and MERTK) have emerged as potential anticancer targets given their roles in regulating innate immune responses as well as driving tumor growth and proliferation (1–4). Growth arrest specific 6 (GAS6) and Protein S are the major and most extensively characterized ligands for these kinases (2, 5). Both GAS6 and Protein S are γ-carboxylated proteins that bind to phosphatidylserine residues on the surface of apoptotic cells through their amino terminal Gla domains, whereas the carboxy domains of GAS6 and Protein S bind to the extracellular domains of the TAM kinases. Thus, GAS6 and Protein S function as bridge proteins for apoptotic cells and the TAM kinases, resulting in efferocytosis. This function of the TAM kinases to mediate apoptotic cell clearance acts to inhibit immunity. The process of efferocytosis mediated by TAMs maintains macrophages in an M2 polarized state and reduces expression of cytokines induced by M1-like macrophages (6). Further, this process clears antigens that may be exposed during cell death, and this may limit the immune response to neoantigens (5). AXL expressed on dendritic cells has also been shown to inhibit immune cell activity. AXL functions together with the type I interferon receptor to increase suppressor of cytokine signaling 1 (SOCS1) and SOCS3 expression, resulting in the termination of inflammatory toll-like receptor signaling (7). PROS1 expression is greatly increased in activated T cells, and PROS1 activates both AXL and MERTK on dendritic cells to attenuate T-cell activation (1). Loss of any member of the TAM family leads to autoimmunity in a gene dosage–dependent manner, consistent with the concept that TAM activation dampens the immune response (2, 3, 8, 9).

The TAM kinases also play a well-studied role in maintaining tumor cell growth, proliferation, and survival. In tumor cells, these kinases induce downstream signaling pathways including PI3K-AKT, RAF-MAPK, and PLC-PKC (10, 11). In this regard, activation of AXL contributes to several fundamental mechanisms of malignancy by promoting cancer cell migration and invasion, enhancing tumor angiogenesis, and facilitating cancer cell survival and tumor growth (11–13). Both AXL and MERTK overexpression and activation are critical mediators of acquired resistance to both conventional chemotherapies and targeted therapies (13). In many cancer types, overexpression and activation of AXL is generally correlated with malignant progression and poor prognosis (12, 14). AXL has been identified as a marker for tumors that have undergone epithelial–mesenchymal transition, and inhibition of AXL has been shown to reverse epithelial–mesenchymal transition (15, 16). MERTK is amplified in a small percentage of esophageal carcinomas and is overexpressed in the absence of gene amplification in many cancers, including multiple myeloma, gastric, prostate, breast, melanoma, and rhabdomyosarcoma (5, 14). Silencing of the *MERTK* gene increased apoptosis, decreased colony formation, increased chemo-sensitivity, and decreased tumor growth in human non-small cell lung cancer models (11).

Based on the clear roles of AXL and MERTK in both immunosuppression and tumor-promoting mechanisms, we set out to develop a potent and selective small molecule dual inhibitor of both AXL and MERTK. Here we demonstrate that INCB081776, currently in phase 1 clinical trials, is a highly selective inhibitor of AXL and MERTK (17) and functions to inhibit AXL and MERTK through immunomodulatory as well as tumor-directed signaling mechanisms. We demonstrate that INCB081776 cooperates with checkpoint blockade to increase functional cytokine production as well as antitumor efficacy in syngeneic models. Lastly, we demonstrate antitumor activity of INCB081776 in sarcoma patient–derived xenograft (PDX) models.

## Materials and Methods

### Cell lines and Reagents

H1299 cells (RRID: CVCL_0060) were maintained in RPMI-1640 (RPMI, ThermoFisher Scientific, Carlsbad, CA, USA; #11875-093) culture medium with 10% fetal bovine serum (FBS; GE Healthcare, Chicago, IL, USA; #SH30071.03) and obtained from American Type Culture Collection (#CRL-5803). Ba/F3 cells were obtained from German Collection of Microorganisms and Cell Cultures (DSMZ; Braunschweig, Germany; #ACC 300) and grown in RPMI supplemented with 10% FBS plus 4 ng/ml interleukin-3. G361 cells (RRID: CVCL_1220) were obtained from American Type Culture Collection (#CRL-1424) and maintained in RPMI medium containing 10% FBS. All human cell lines have been authenticated using short tandem repeat profiling within the last 3 years. All experiments were performed with mycoplasma-free cells. INCB081776 was produced by Incyte Corporation (ClinicalTrials.gov identifier #NCT03522142). Human peripheral blood mononuclear cells (PBMCs) were obtained from normal leukapheresis of two healthy donors (Biological Specialties, Colmar, PA, USA).

### Biochemical Enzyme Assays, ATP Competition Assay, and Kinase Profile Assays

Details of standard biochemical enzyme, ATP competition, and kinase profile assays conducted in our study are provided in the **Supplementary Methods**.

### Cell Proliferation Assays

The cytoplasmic domain of AXL, MERTK, or TYRO3 fused with dimerization sequence and HA tag was cloned into a pMSCV (murine stem cell virus) vector with a puromycin-resistance marker to generate three constructs individually by electroporation into Ba/F3 cells. Single clones that were interleukin-3−independent and puromycin-resistant were selected and characterized. To evaluate effects on Ba/F3 cell proliferation, 1,000 cells/well of Ba/F3, Ba/F3-AXL, Ba/F3-MERTK, or Ba/F3-TYRO3 cells were treated in the presence or absence of INCB081776 at various concentrations (10 concentration points with a three-fold dilution factor from the highest concentration of 10 µM) diluted in RPMI with 2% FBS for 48 h in a 384-well plate. Cell viability was measured by ATP assay (CellTiter-Glo Assay, Promega, Madison, WI, USA) according to the manufacturer’s procedure. The data were converted to percent inhibition relative to dimethyl sulfoxide (0.4% DMSO) control, and half maximal inhibitory concentration (IC_50_) curves were fitted using GraphPad Prism software (San Diego, CA, USA).

### pAXL Inhibition Assay in H1299 Cells

To measure the effect of INCB081776 on pAXL, H1299 cells were plated (30,000 cells/well) in 96-well tissue-culture plates (Costar, Corning Incorporated, Corning, NY, USA) and incubated overnight at 37°C with 5% CO_2_. INCB081776 at an appropriate concentration was added and incubated for 1 h at 37°C with 5% CO_2_. rhGAS6 (R&D Systems, Minneapolis, MN, USA; #885-GSB) was added at 1 µg/ml to each well, and plates were incubated at 37°C with 5% CO_2_ for 15 min. Cells were harvested and lysed in 110 µl of ice-cold lysis buffer (Cell Signaling Technology, Danvers, MA, USA; #9803) with protease and phosphatase inhibitors (ThermoFisher Scientific; #78446) for 1 h on ice and stored at −80°C for ELISA. ELISA plates were prepared by incubating Greiner lumitrac high-binding plates with 8 µg/ml of anti-AXL antibody (R&D Systems; MAB154) overnight at room temperature. The plates were washed and blocked with phosphate-buffered saline (PBS) with 0.1% bovine serum albumin. Cell lysates were loaded onto ELISA plates and incubated 2 h at room temperature. The plates were washed and incubated with LANCE Eu-W1024 anti-phospho-tyrosine antibody (Perkin Elmer, Waltham, MA, USA; #AD0067) in DELFIA assay buffer (Perkin Elmer; #4002-0010) for 2 h at room temperature, washed, and DELFIA Enhancement Solution (Perkin Elmer; #4001-0010) was added. The plates were gently shaken for 15 min at room temperature and read on the PheraStar (BMG Labtech, Ortenberg, Germany). The data were converted to percent inhibition relative to DMSO control, and INCB081776 IC_50_ determination was performed by fitting the curve of percent inhibition versus the log of the inhibitor concentration using GraphPad Prism.

### Phospho-MERTK Inhibition Assay in G361 Cells

Newly thawed G361 cells were allowed to recover for three passages before use, and only cells within 20 passages after thawing were used in the assay. Cells were kept under nonconfluent conditions and used in log-phase growth. Two milliliters of 1 × 10^6^ cells/ml (2 × 10^6^ cells/well) G361 cells were added to six-well tissue-culture plates (Corning Incorporated; #3961) for 2 days. At the time of the assay, 1 ml of medium was added to each well. To determine the activity of INCB081776, a stock solution of 5 mM INCB081776 in DMSO was used to make three-fold serial dilution of DMSO working stocks that were further diluted in culture medium, and 100 μl of the diluted compound was added to each well with final concentrations ranging from 0.2 nM to 1 μM. For control wells in the absence of INCB081776, 100 μl of 0.22% DMSO was added to maintain the final 0.02% DMSO concentration in every sample. The mixtures of cells and compound were incubated for 1 h at 37°C in a humidified incubator supplemented with 5% CO_2_, then 10 μl of 55.5 μg/ml of MERTK-activating antibody (R&D Systems; #MAB8912; final concentration equal to 500 ng/ml) in PBS was added to each well, except the unstimulated sample, and incubated for 30 min at 37°C in a humidified incubator supplemented with 5% CO_2_. After incubation, each well was washed twice with 2 ml of cold PBS. Lysis buffer (120 μl; Cell Signaling Technology; #9803) containing 1 mM PMSF, Halt phosphatase inhibitors (1:100 dilution; ThermoFisher Scientific; #78426), and protease inhibitors (1:50 dilution; Calbiochem^®^ #535140; MilliporeSigma, Burlington, MA, USA) was added to each sample and incubated on ice for 30 min. The cell extracts were transferred to a 96-well V bottom plate, centrifuged at 3000 rpm for 10 min at 4°C, and the extracts were stored at 80°C until analysis by ELISA for phospho-MERTK (pMer; R&D Systems; #DYC2579). The optical density of the plate was measured using a Molecular Devices SpectraMax Plus microplate reader (Molecular Devices, San Jose, CA, USA) at 450 nm with wavelength correction at 540 nm. Absorbance of the standards was plotted versus the concentration to generate a standard curve using four-parameter algorithm curve-fitting software (SOFTmax PRO application, Molecular Devices). pMERTK concentrations for unknown samples were determined by extrapolation from the standard curve. IC_50_ values were calculated by GraphPad Prism 7.0 using a nonlinear regression sigmoidal dose-response curve with variable slope.

### Phagocytosis Assay

Peripheral blood of healthy donors was procured from BioIVT (Westbury, NY, USA). A stock concentration of 5 mM INCB081776 in DMSO was diluted in PBS to concentrations of 10 nM and 100 nM. Blood was incubated for 19 h with INCB081776 at concentrations of 10 nM and 100 nM along with a DMSO control. On the next day, blood was diluted with an equal volume of PBS and 2% FBS, and PBMCs were isolated using Lymphoprep and SepMate-50 tubes (STEMCELL Technologies, Vancouver, BC, Canada) in accordance with the manufacturer’s recommendations. The cells were counted, washed once in filtered PBS, and then were re-suspended in serum-free Na^+^ medium (145 mM NaCl, 5 mM KCl, 10 mM HEPES, 0.1% bovine serum albumin, 5 mM D-glucose, pH 7.5, at 37°C). The cells were then stained with APC conjugated anti-CD14 and BV786 conjugated anti-CD16 (BD Biosciences, San Jose, CA, USA) in Na^+^ medium for 30 min at room temperature. After 30 min, the cells were washed and re-suspended in Na^+^ medium. PBMCs (2 × 10^6^ cells in a volume of 100 μl Na^+^ medium) pre-labeled with APC anti-CD14 and BV786 anti-CD16 mAb were added to 900 μl pre-heated (37°C) Na^+^ medium, vortexed, and flow cytometry acquisition was performed on BD LSRFortessa X-20 (BD Biosciences) for 20 s. To measure the bead uptake, 1 μl of Fluoresbrite Yellow-Green latex microspheres (Polysciences, Warrington, PA, USA) was added and the mixture vortexed prior to acquisition for 20 s. After 20 s, the tube was vortexed again and acquisition was performed for 20 s. This process of mixing the cells followed by acquisition was repeated until 4 min elapsed. Cells were gated by forward and side scatter and by CD14 APC, CD16 BV786 fluorescence signals. The mean fluorescence intensity of the Yellow-Green latex microspheres in CD14^++^CD16^+^ cells over successive 20-s intervals was plotted against time.

### Inhibition of MERTK Activity in Primary Macrophages

PBMCs were separated using Ficoll–Paque density gradient centrifugation and any remaining red blood cells were lysed using 1 × RBC Lysis Buffer (Cell Signaling Technology) for 5 min at room temperature. The PBMCs were washed with PBS before being enriched for monocytes using CD14 microbeads positive selection separation following the manufacturer’s protocol as specified (AutoMacs Pro, Miltenyi Biotec, Bergisch Gladbach, Germany). The CD14^+^ cells were initially seeded at 1.5 × 10^6^ per well in six-well plates in RPMI + 10% heat-inactivated FBS and 10% AB human serum (Sigma-Aldrich Corp., St Louis, MO, USA), 100 U/ml penicillin + 100 μg/ml streptomycin (Corning), supplemented with 100 ng/ml macrophage colony-stimulating factor (R&D Systems; #216-MC) and were cultured at 37°C, 5% CO_2_ for 10 days. Fresh macrophage colony-stimulating factor was added to the media every 3 days until the macrophages had attached. In preparation for the assay, the media was removed and the cells were re-fed with fresh media without human serum. INCB081776 stocks were prepared at 1000× in 100% DMSO and diluted 67-fold first into media and then a further 15-fold when added to the macrophages. The macrophages were treated with INCB081776 for 2 h at 37°C, 5% CO_2_. Five μg/ml anti-MERTK antibody MAB8912 (R&D Systems) was added to the macrophages for an additional 30 min, at which time the cells were washed with cold PBS. All PBS was carefully aspirated from the wells and the dry plates were frozen at −20°C.

### Western Blotting

Macrophages were allowed to thaw on ice before being lysed with 250 μl/well of 1× Lysis Buffer (Cell Signaling Technology; #9803) and Halt protease and phosphatase inhibitors (ThermoFisher Scientific) for 1 h at 4°C. The lysed cells were scraped and transferred to an Eppendorf vial on ice. The lysates were centrifuged at 12,700 rpm for 15 min at 4°C. H1299 and PDX tumors were weighed and homogenized with lysis buffer supplemented with protease and phosphatase inhibitor cocktails (Roche, Basel, Switzerland; #11836170001). Tumors were lysed on ice for 30 min, followed by centrifugation at 13,000 rpm for 10 min. Protein lysates were quantified using BCA Protein Assay Kit (Pierce, ThermoFisher Scientific; #23225). Lysates were transferred to a new tube together with 6× Laemmli SDS sample buffer (Alfa Aesar, Haverhill, MA, USA) and the samples were heated for 6 min at 95°C. Approximately 50 μg of protein sample was loaded per well using Novex™ 8%–16% Tris-Glycine Mini Gels or 4%–12% Tris-Glycine Novex WedgeGels (Invitrogen, Carlsbad, CA, USA). The proteins were transferred to a nitrocellulose membrane using an iBlot (ThermoFisher Scientific) dry blotting system. The membranes were blocked with 0.5% nonfat dry milk in wash buffer (100 mM NaCl, 10 mM Tris-HCl, pH 8.0, 0.1% Tween 20) for 1 h at room temperature. The primary antibody used for pMERTK was from PhosphoSolutions (Aurora, CO, USA; #p186-749). The remaining antibodies were obtained from Cell Signaling Technology: MERTK (#4319), phosphor-AXL (#5724), AXL, (#4566), GAS6 (#67202), phosphor-AKT (#4060), AKT (#9272), and β-Actin (#4970). Primary antibodies were added at 1:500 and 1:1,000, respectively, in 0.5% milk/wash buffer and rocked overnight at 4°C. The membranes were washed three times in wash buffer before incubation with the secondary antibody (anti-rabbit IgG1-HRP; Cell Signaling Technology) at 1:2500 in 0.5% milk/wash buffer for 2 h at room temperature. The membranes were washed again before the bands were detected with SuperSignal West Dura Extended Duration Chemiluminescent substrate (ThermoFisher Scientific) and were visualized using the Fluorochem M Digital Imager (Protein Simple, San Jose, CA, USA).

### Macrophage Suppression of T-Cell Proliferation Assay

Human PBMCs were isolated from the peripheral blood of healthy donors by density gradient centrifugation on Ficoll-Hypaque (GE Healthcare, Chicago, IL, USA; #17-1440-02) followed by purification with anti-CD14 Microbeads (Miltenyi Biotec; #130-050-201). Isolated CD14^+^ monocytes/macrophages were incubated with 100 ng/ml macrophage colony-stimulating factor (R&D Systems; #216-MC) and 50 ng/ml TGFβ1 (R&D Systems; #240-B) at 37°C for 6 days; 100 μl/well of CD14^+^ macrophages were seeded at 0.5 × 10^6^ cells/ml in a 96-well round-bottomed culture plate (Costar; #3799) and treated with INCB081776 overnight at 37°C. CD4^+^CD25^−^ effector T (T_eff_) cells were isolated using the Dynabeads regulatory CD4^+^CD25^+^ T-cell kit (Life Technologies, Carlsbad, CA, USA; #11363D) and the T_eff_ cells were labeled with carboxyfluorescein succinimidyl ester (CFSE) using the CellTrace CFSE Cell Proliferation kit (ThermoFisher Scientific; #C34554). Freshly CFSE-labeled T_eff_ cells were mixed with Dynabeads Human T-Activator CD3/CD28 (ThermoFisher Scientific; #11132D) at a ratio of 5:1, then 100 μl/well of the T-cells/beads mixture at 1 × 10^6^ cells/ml were added to the INCB081776-treated CD14^+^ macrophages and continue to culture at 37°C for 5 days. T_eff_ cells were analyzed by a flow cytometer (BD LSRFortessa X-20, BD Biosciences) and the cell-free supernatant was tested in the Luminex assay (Millipore; #HCYTOMAG-60K_38plex) to measure the concentrations of different cytokines and chemokines.

### *In Vivo* Efficacy Studies

MBT-2 mouse bladder carcinoma cells were obtained from the Japanese Collection of Research Bioresources Cell Bank and were maintained in DMEM supplemented with 10% FBS. MC38 cells were obtained from the National Cancer Institute. 4T1 cells were obtained from American Type Culture Collection and maintained in RPMI media supplemented with 10% FBS. For MBT-2 experiments, 5 × 10^5^ MBT-2 cells were inoculated subcutaneously into the right hind flank of 6- to 8-week-old C3H mice or athymic nude mice (Charles River Laboratories, Wilmington, MA, USA). For MC38 studies, 12-week-old female C57BL/6 mice were inoculated with brei of MC38 tumors (*in vivo* passage 6) into the right hind flank. For 4T1 studies, 7.5 × 10^5^ 4T1 cells were inoculated subcutaneously into the right hind flank of 6- to 8-week-old female BALB/c mice or athymic nude mice (Charles River Laboratories). Mice were randomized by tumor volume with *n* = 10–12 per group using Study Director software (Studylog Systems, South San Francisco, CA, USA). INCB081776 was dissolved in 50 mM citrate buffer (Alfa Aesar, Ward Hill, MA, USA; #J63008AP) in 0.5% methylcellulose (Sigma-Aldrich Corp.; #M0430) and was dosed orally twice a day (BID) continuously from the start to the end of study. In the MC38 study, anti–programmed death ligand 1 (PD-L1) (Bio X Cell, West Lebanon, NH, USA; BE0101) was dosed intraperitoneally at 15 mg/kg twice a week. The vehicle group and INCB081776 group were also administered rat IgG2b control antibody twice a week (BE0090, Bio X Cell) beginning at the start of treatment. In the 4T1 study, a single dose of anti–PD-L1 was dosed intraperitoneally at 15 mg/kg at the start of the study. In the combination studies, the vehicle control group and mice receiving anti–PD-L1 were dosed with vehicle BID continuously until the end of study. Vehicle and INCB081776 were dosed orally BID. Mice were monitored for tumor growth and overt tolerability over the course of the experiment. PDX tumor models were conducted at Champions Oncology (Hackensack, NJ, USA). PDX tumors were implanted into 6- to 8-week-old female athymic nude mice. When tumor volumes were approximately 150–250 mm^3^, mice were randomized by tumor volume and were administered INCB081776 at 30 mg/kg BID by oral gavage. Tumor volume was calculated using the formula (L × W^2^)/2, where L and W refer to the length and width dimensions, respectively. Tumor growth inhibition was calculated using the formula (1 − (V_T_/V_C_)) × 100, where V_T_ is the tumor volume of the treatment group on the last day of treatment, and V_C_ is the tumor volume of the control group on the last day of treatment. Two-way analysis of variance with Dunnett’s multiple comparisons test was used to determine statistical differences between treatment groups (GraphPad Prism).

Mice were housed at 10–12 animals per cage, and were provided enrichment and exposed to 12-h light/dark cycles. The number of mice used per treatment group was based on historical experience with each tumor model. Mice whose tumor volumes exceeded limits (10% of body weight) were humanely euthanized by CO_2_ inhalation. Animals were maintained in a barrier facility fully accredited by the Association for Assessment and Accreditation of Laboratory Animal Care, International. All of the procedures were conducted in accordance with the US Public Service Policy on Human Care and Use of Laboratory Animals and with Incyte Animal Care and Use Committee Guidelines.

### Pharmacokinetic Studies

On the last day of the MBT-2 efficacy study in C3H mice (day 18) or nude mice (day 14), plasma was collected at 1, 2, 4, 8, and 16 h (C3H mice) or 2, 4, and 16 h (nude mice) after the last oral administration of INCB081776. Samples were collected in EDTA-coated microtubes (Greiner Bio-one, Kremsmunster, Austria; 450475). Plasma concentrations of INCB081776 were determined with a calibration curve prepared in plasma. The assay range was 1–5,000 nM. Study sample aliquots (25 µl) were deproteinized with vigorous mixing with six volumes of acetonitrile (150 µl) containing INCB073305 as the internal standard. After centrifugation, 100 µl of the supernatants were transferred to a 96-well plate containing 200 µl of water, mixed well, and analyzed by liquid chromatography–tandem mass spectrometry. Chromatography was performed using 2-µl injections of extracts with an ACE C18-AR, (30 × 2.1 mm, 3 μm, at 40°C) column under gradient conditions at a flow rate of 0.75 ml/min. 0.1% formic acid in water and 0.1% formic acid in acetonitrile were used for mobile phases A and B, respectively. Liquid chromatography–tandem mass spectrometry analysis was performed using a Shimadzu Nexera UHPLC system coupled to the electrospray ionization source (in positive ion mode) of a Sciex 6500+ triple quadrupole mass spectrometer. Peak areas for the multiple reaction monitoring transitions 636.3 > 121.0 for INCB081776 and 695.3 > 636.1 for the internal standard were used to calculate Analyte/internal standard peak area ratios, which were then used to create linear regression calibration equations with 1/x^2^ weighting.

### Immunophenotyping and Cytokine Quantitation

At the end of study, tumors were collected 4 h after the last dose of INCB081776 and placed on ice. Tumor samples were cut into 2-mm pieces and transferred to Miltenyi C Tubes (Miltenyi Biotec; #130-096-334). Tumor dissociation was conducted according to the manufacturer’s protocol (Miltenyi Biotec; #130-096-730). The filter was rinsed with cold PBS and samples pelleted. Red blood cells were lysed in Pharm Lyse (BD; #555899). Cells were washed with PBS and re-suspended in PBS with Live/Dead stain (LifeTech Scientific, Shenzhen, China; #L34966) for 15 min at room temperature. Cells were then washed in PBS and re-suspended in stain buffer (BD; #554657), and the following antibodies were added to the samples for 30 min at 4°C: CD3 (BD; #553062), CD45 (BD; #564279), CD8 (BD; #560182), CD4 (BD; #552775), CD11b (BD; #557657), F4/80 (eBiosciences, San Diego, CA, USA; #12-4801-82), MHC Class II (BD; #562564), and CD206 (BioLegend, San Diego, CA, USA; #141708). Cells were then washed, fixed, and permeabilized with fixation/permeabilization buffer (eBioscience 00-5523-00). Cells were re-suspended in permeabilization buffer (eBioscience; 00-8333). Ki-67 antibody (BioLegend; #652413) was added to the samples for 1 h at room temperature. Cells were then washed and re-suspended in stain buffer (BD; #554657) for acquisition. M1 macrophages were identified within the CD45^+^, CD11b^+^ F4/80^+^ population as MHC Class II^Hi^/CD206^Lo^ and M2 macrophages were identified as MHC Class II^Lo^/CD206^Hi^. Data were acquired on a BD Fortessa and analyzed with FlowJo software. Levels of interferon (IFN)-γ within tumors were quantitated with a multiplexed protein detection kit according to the manufacturer’s protocol (MesoScale Diagnostics, Rockville, MD, USA). Statistical significance was determined by one-way analysis of variance with Dunnett’s multiple comparisons test (GraphPad Prism).

## Results

The biochemical potency of INCB081776 to inhibit the enzymatic activity of TAM family members was investigated by time-resolved fluorescence energy transfer assays using recombinant phosphorylated forms of the kinase domains for AXL, MERTK, and TYRO3. The average IC_50_ values from multiple lots of INCB081776 against AXL, MERTK, and TYRO3 were 0.61 ± 0.31 nM (*n* = 18), 3.17 ± 1.97 nM (*n* = 25), and 101 ± 27 nM (*n* = 25), respectively, demonstrating approximately 30-fold selectivity over TYRO3 (**Table 1** **)**. INCB081776 was also evaluated at 200 nM in a comprehensive kinase study, which included 179 kinases. INCB081776 was approximately 60-fold selective for AXL and MERTK compared with c-MET and did not inhibit any additional kinases (**Supplementary Table 1**). These results demonstrate that INCB081776 is a potent and highly selective inhibitor of AXL and MERTK. The mode of inhibition with respect to ATP concentration was evaluated using a MERTK assay. The IC_50_ values of INCB081776 for MERTK increased linearly with ATP concentration, indicating an ATP-competitive mode of inhibition (**Supplementary Figure 1**).

**Table 1:** Biochemical potency of INCB081776 against AXL, MERTK, and TYRO3.

Recombinant kinase	Biochemical IC50,Mean ± SD (nM)	Number of replicates (n)
AXL	0.61 ± 31	18
MERTK	3.17 ± 1.97	25
TYRO3	101 ± 27	25

**Figure 1:**
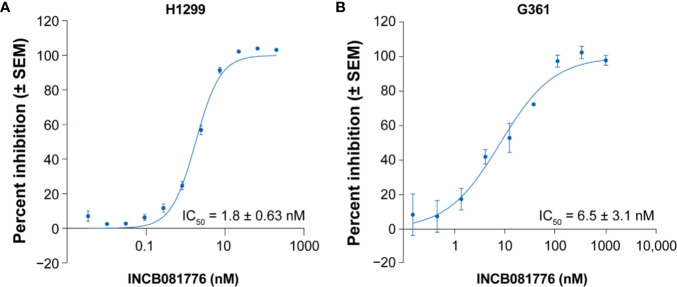
Inhibition of pAXL and pMERTK in tumor cell lines. **(A)** H1299 cells were pretreated with INCB081776 for 1 h, then stimulated with recombinant human GAS6 for 15 min. Cell lysates were quantified for pAXL by ELISA (*N* = 19). **(B)** G361 cells were pretreated with INCB081776 for 1 h followed by 30 min of incubation with a MERTK agonist antibody, MAB8912 (*N* = 4). Levels of pMERTK were quantified by ELISA. Half maximal inhibitory concentration (IC_50_) values were determined by fitting the curve of percent inhibition versus the log of INCB081776 concentrations using sigmoidal dose response with variable slope in GraphPad Prism. SEM, standard error of the mean.

To evaluate the cellular potency and selectivity within the TAM receptor family, mouse Ba/F3 cell lines with stable expression of AXL, MERTK, or TYRO3 were generated. Treatment of stable Ba/F3 transfectants with INCB081776 potently inhibited the proliferation of Ba/F3 cells expressing either AXL or MERTK with concentration required for 50% growth inhibition values of 16 ± 11 nM (*n* = 52) and 14 ± 4.9 nM (*n* = 65), respectively, but weakly inhibited the growth of TYRO3-expressing Ba/F3 cells (IC_50_ = 498 ± 161 nM; *n* = 59) and was inactive against parental Ba/F3 cells (IC_50_ >4000 nM) (**Table 2**). These cellular data are consistent with the biochemical data and confirm that INCB081776 is a potent inhibitor of AXL and MERTK, and is >30-fold more selective for AXL and MERTK than TYRO3. In addition to engineered recombinant cell lines, the ability of INCB081776 to modulate AXL and MERTK activity was evaluated in tumor cell lines expressing high levels of endogenous AXL or MERTK. The non-small cell lung cancer cell line H1299 has been shown to exhibit markedly increased AXL protein expression (18). INCB081776 treatment of H1299 cells potently inhibited pAXL with an IC_50_ value of 1.8 ± 0.63 nM (*N* = 19, **Figure 1A**). Similarly, the potency of INCB081776 in blocking MERTK autophosphorylation was evaluated in G361 cells, a melanoma cell line expressing high level of MERTK (19). As shown in **Figure 1B**, INCB081776 effectively blocked MERTK phosphorylation induced by MAB8912 in G361 melanoma cells with an IC_50_ value of 6.5 ± 3.1 nM (*N* = 4).

**Table 2:** Cellular potency of INCB081776 against AXL, MERTK, and TYRO3.

Ba/F3 cell line	BAF3 growth inhibition IC_50_,mean ± SD (nM)	Number of replicates (n)
AXL	16 ± 11	52
MERTK	14 ± 4.9	65
TYRO3	498 ± 161	59
WT (no kinase)	>4,000	11

IC_50_, concentration required for 50% growth inhibition; SD, standard deviation.

**Figure 2:**
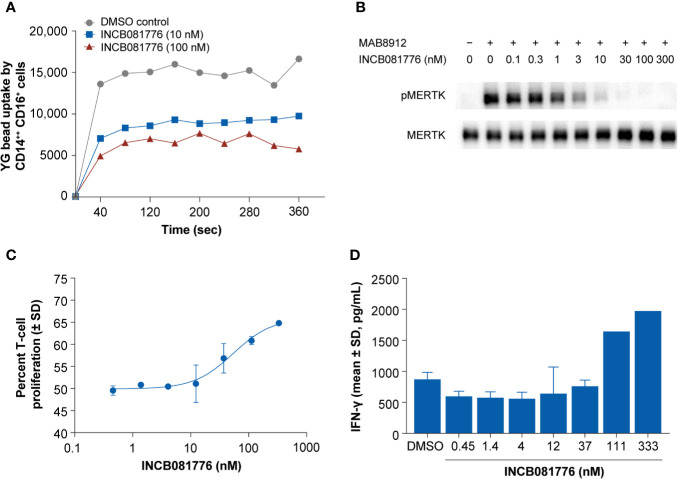
Inhibition of phagocytosis and reversal of macrophage-mediated suppression of T cells by INCB081776 in primary human immune cells. **(A)** Human whole blood was treated with INCB081776 at 10 nM and 100 nM along with dimethyl sulfoxide control. Fluorescent microspheres were added to peripheral blood mononuclear cells (PBMCs) prelabeled with anti-CD14 and anti-CD16, and acquisition performed at intervals over a course of 4 min. Plot indicating the mean of the uptake of fluorescent microspheres by CD14^++^CD16^+^ monocyte population over time is shown. Data are representative of three healthy donors. **(B)** Primary macrophages differentiated *in vitro* from human PBMCs were pretreated for 2 h with INCB081776 followed by stimulation with the MERTK-specific agonist antibody MAB8912 for an additional 30 min. Levels of phospho-MERTK (pMERTK) and total MERTK were determined by Western blot. **(C)** Primary macrophages differentiated *in vitro* from human PBMCs were pretreated with various concentrations of INCB081776 overnight, followed by incubation with carboxyfluorescein succinimidyl ester (CFSE)-labeled T cells for an additional 5 days. T-cell proliferation was determined by FACS analysis (*N* = 2 per group). **(D)** Following incubation of macrophages and T cells in the presence of increasing concentrations of INCB081776 as in panel **(C)**, supernatants were collected and interferon (IFN)-γ cytokine production was measured (*N* = 2 per group, except for 111 and 333 nM [*N* = 1]). SD, standard deviation.

The TAM family of kinases play a key role in efferocytosis. To study the role of INCB081776 in inhibiting phagocytic uptake, a phagocytosis assay using primary human monocytes was employed (**Supplementary Figure 2**) (20). Analysis of AXL and MERTK expression in intermediate (CD14++CD16+) and classical (CD14++CD16-) monocytes from healthy donors revealed that intermediate monocytes had higher expression of MERTK than classical monocytes, consistent with a previous report (21) (**Supplementary T 3A**). Both cell populations also lacked AXL expression (**Supplementary Figure 3B**) (21). INCB081776 reduced microsphere uptake in both intermediate and classical monocytes in a concentration-dependent manner (**Figure 2A** and **Supplementary Figure 3C**). These data demonstrate that INCB081776 reduced phagocytic ability of intermediate and classical monocytes, likely through inhibition of MERTK.

It is thought that MERTK plays a predominant role on the phagocytic ability of immunosuppressive macrophages (6, 22, 23). To explore the effect of INCB081776 in inhibiting MERTK in primary human macrophages, a macrophage-inhibition assay was employed. MAB8912 was discovered to be a MERTK agonist antibody, and addition of this antibody to primary human macrophages led to MERTK phosphorylation (**Figure 2B**). Pretreatment of macrophages with INCB081776 prior to agonism with MAB8912 blocked the induction of phosphorylated MERTK in a concentration-dependent manner with an IC_50_ of 1.6 ± 0.4 nM (**Figure 2B**). These data suggest that INCB081776 can inhibit MERTK activity in primary human immune cells. Recent work on monocyte-derived macrophages suggests that these macrophages may exhibit immunosuppressive function and can act as suppressor cells that inhibit T-cell proliferation (24). To examine the functional activity of INCB081776, the effects on macrophage-mediated suppression of T-cell proliferation was evaluated. **Figure 2C** demonstrates that INCB081776 partially reversed macrophage-mediated suppression of T-cell proliferation. This was associated with an increase in IFN-γ production, suggesting that IFN-γ release was a consequence of the increased T-cell proliferation (**Figure 2D**).

To explore the activity of INCB081776 *in vivo*, pharmacodynamic studies were initially performed to determine dose levels required for target inhibition. For these studies, the modulation of phosphorylated AXL in the H1299 tumor model was chosen. H1299 tumor-bearing SCID mice were dosed with INCB081776 at dose levels of 3, 10, or 30 mg/kg, and tumors were evaluated for pAXL and total AXL expression. The 3 mg/kg dose level appeared to partially inhibit pAXL, whereas full inhibition was observed at the 10 and 30 mg/kg dose levels (**Supplementary Figure 4A**). A time course study was then performed where H1299 tumors were collected at various timepoints after tumor-bearing mice were administered a single 30 mg/kg dose of INCB081776. **Supplementary Figure 4B** demonstrates that pAXL was fully inhibited up to 8 h post-dose; however levels rebounded at 16h. These data suggested that twice daily dosing was appropriate for dosing INCB081776 in antitumor efficacy studies (18).

**Figure 3:**
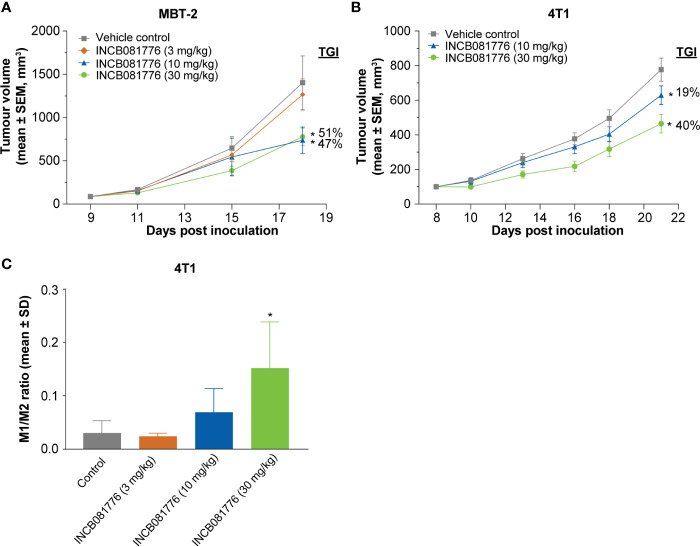
Antitumor tumor activity of INCB081776 is dependent on the immune system in syngeneic models. **(A)** C3H mice bearing established MBT-2 tumors were administered INCB081776 orally twice a day at the indicated dose levels. *N* = 12 mice per group. Tumor growth inhibition values are indicated. **(B)** BALB/c mice bearing established 4T1 tumors were dosed with INCB081776 at 10 and 30 mg/kg orally twice a day. *N* = 12 per group. Statistical analysis was performed using two-way analysis of variance with Dunnett’s multiple comparisons test in panels **(A, B)**. *p < 0.05 compared with control. **(C)** Immunophenotyping of MBT-2 tumor–bearing mice treated with INCB081776 showed a dose-related increase in the M1-/M2-like macrophage ratio. *N* = 4 per group. Statistical analysis was performed using one-way analysis of variance with Dunnett’s multiple comparisons test *p < 0.05 compared with control. SD, standard deviation; SEM, standard error of the mean; TGI, tumor growth inhibition.

**Figure 4:**
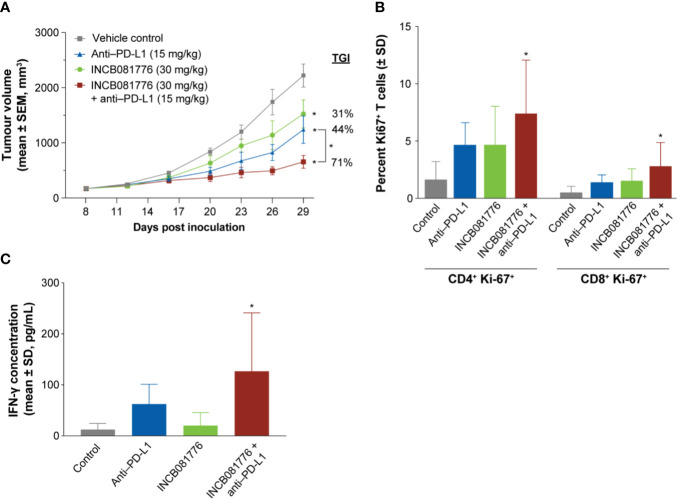
INCB081776 displays enhanced antitumor activity in combination with checkpoint blockade. **(A)** C57BL/6 mice bearing established MC38 tumors were dosed orally twice a day with INCB081776, anti–programmed death ligand 1 (PD-L1) twice a week, or the combination. *N* = 12 mice per group. Statistical analysis was performed using two-way analysis of variance with Dunnett’s multiple comparisons test. *p < 0.05 compared with control and for the between group comparison. **(B)** The percentage of CD4^+^Ki67^+^ and CD8^+^Ki67^+^ cells were determined following treatment of MC38 tumor–bearing mice with INCB081776, anti–PD-L1, or the combination. **(C)** Levels of interferon (IFN)-γ in tumor extracts from mice at end of study were determined. *N* = 5 per group in panels **(B, C)**. Statistical analysis was performed using one-way analysis of variance with Dunnett’s multiple comparisons test in panels **(B, C)**. *p < 0.05 compared with control. SD, standard deviation; SEM, standard error of the mean; TGI, tumor growth inhibition.

To test potential immunomodulatory activity of INCB081776 *in vivo*, antitumor efficacy studies were performed in syngeneic tumor models, which utilize immunocompetent mice. INCB081776 induced dose-dependent efficacy in the MBT-2 and 4T1 tumor models (**Figures 3A, B**). INCB081776 was well tolerated and no changes in body weight were observed (**Supplementary Figures 5A, B**). INCB081776 was also dosed in nude mice, which lack functional T cells. There was no activity of INCB081776 in these mice, demonstrating the antitumor activity was dependent on T cells, at least in these models (**Supplementary Figures 6A, B**). In the MBT-2 antitumor efficacy studies, plasma was collected at various time points after the last oral dose of INCB081776. Pharmacokinetic analysis revealed dose-dependent plasma levels of INCB081776 in both C3H and nude mice (**Supplementary Figures 7A, 7B**). In the MBT-2 model, INCB081776 treatment increased the ratio of M1-like to M2-like macrophages in a dose-dependent manner (**Figure 3C**). Given the functional reversal of macrophage-mediated T-cell suppression observed *in vitro*, we hypothesized that INCB081776 might cooperate with checkpoint blockade to further boost antitumor responses. In the MC38 model, both INCB081776 and anti–PD-L1 had single-agent antitumor activity, but significantly higher activity in combination (**Figure 4A**). A similar enhanced combination effect was observed in the 4T1 model (**Supplementary Figure 8**). In the MC38 model, both single-agent treatments induced proliferation of CD4^+^ and CD8^+^ tumor-infiltrating lymphocytes, and to a higher degree in combination (**Figure 4B**). Combination treatment also resulted in increased IFN-γ levels in tumors compared with single-agent treatment (**Figure 4C**). These data demonstrate that INCB081776 induced macrophage polarization, increased functional CD4^+^ and CD8^+^ T-cell activity, and combined with checkpoint blockade to enhance antitumor activity *in vivo*.

**Figure 5:**
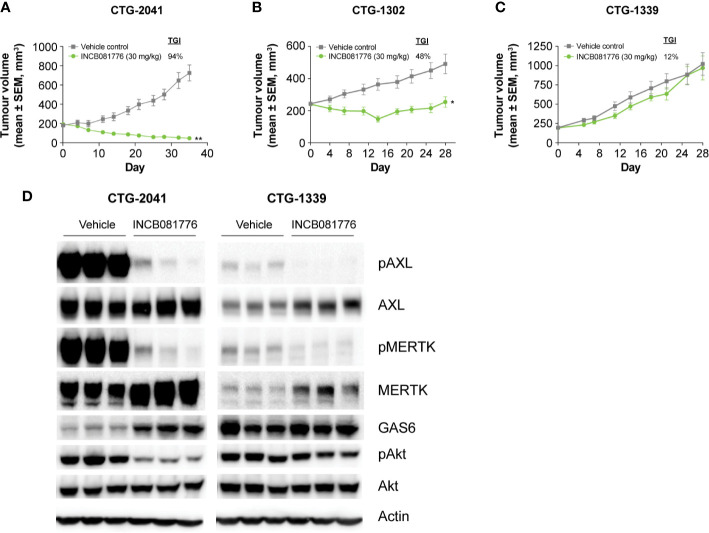
Activity of INCB081776 in sarcoma PDX models. Athymic nude mice bearing **(A)** CTG-2041 **(B)** CTG-1302, or **(C)** CTG-1339 tumors were dosed orally twice a day (BID) with INCB081776 at 30 mg/kg. *N* = 10 mice per group in panels **(A–C)**. Statistical analysis was performed using two-way analysis of variance with Sidak’s multiple comparisons test **(D)** CTG-2041 or CTG-1339 tumors from mice treated with INCB081776 at 30 mg/kg twice a day (BID) or vehicle were lysed and processed by Western blot for pAXL, AXL, pMERTK, MERTK, GAS6, pAKT, AKT, and β-Actin. Three mice per group were analyzed. Western blot data were obtained from different gels. *p < 0.05; **p < 0.0001; compared with control. SEM, standard error of the mean; TGI, tumor growth inhibition.

Besides playing roles in the regulation of immunity, AXL and MERTK provide growth and survival signals to the tumor cells directly (15, 25, 26).Sarcomas are a mesenchymal tumor type where elevated AXL and MERTK expression has been observed (25–27). Based on this, we evaluated INCB081776 for antitumor activity in sarcoma PDX models that expressed AXL and/or MERTK. INCB081776 induced a strong antitumor response in CTG-2041 with 94% tumor growth inhibition (**Figure 5A**). A robust antitumor response was also observed in the CTG-1302 model (**Figure 5B**). In contrast, CTG-1339 was resistant to INCB081776 treatment (**Figure 5C**). Two additional sarcoma PDX models were partial responders and nine others were found to be nonresponsive to INCB081776 (**Supplementary Table 2**). To understand potential mechanisms responsible for the differences observed in responder compared with nonresponder models, we evaluated the impact of INCB081776 on AXL and MERTK activation as well as downstream signaling in CTG-2401 and CTG-1339 tumors (**Figure 5D**). Whereas INCB081776 inhibited pAXL and pMERTK in both models, CTG-2041 expressed significantly higher levels of total AXL and MERTK, especially pAXL and pMERTK (**Figure 5D**). These data imply that AXL and MERTK are strongly activated in CTG-2041. Interestingly, INCB081776 only inhibited pAKT in the CTG-2041 model, which correlated with the antitumor efficacy. INCB081776 treatment also increased total MERTK levels in both tumor models, as well as GAS6 levels in CTG-2041. In total, these data suggest that INCB081776 can inhibit pAXL and pMERTK in sarcoma PDX tumors and that pAKT inhibition correlated with antitumor efficacy.

## Discussion

AXL and MERTK kinases are increasingly being pursued as anticancer drug targets (28). The appeal of these kinases as anticancer targets stems from their roles in promoting tumor growth *via* both immunosuppressive and tumor-promoting mechanisms (3, 5, 29). Based on these features, we developed a highly specific, dual inhibitor of AXL and MERTK. Selectivity data confirm that INCB081776 is selective and is equipotent for both AXL and MERTK. This dual targeting may be important in the design of small molecule inhibitors of the TAM family. It has been widely reported that AXL is a mediator of resistance to endothelial growth factor receptor inhibitors, as well as other targeted therapies and chemotherapies (28, 30, 31). Recently, it was shown that MERTK is upregulated following genetic silencing or pharmacologic inhibition of AXL (32). Dual targeting of AXL and MERTK led to a more potent inactivation of downstream signaling and reduced tumor growth *in vivo* compared with targeting either kinase alone (32). These data highlight the possibility that a dual inhibitor of AXL and MERTK may circumvent resistance mechanisms that cells may develop to AXL inhibition. An additional report describing the pan-TAM inhibitor RXDX-106 concluded that inhibition of TYRO3 did not play a major role in the antitumor activity of the compound, supporting the idea that inhibition of AXL and MERTK are the most relevant targets in the TAM family (33).

It is believed that MERTK plays a predominant role in efferocytosis of M2-like macrophages, which produces immunosuppressive signals (6, 22, 23). Studies have previously shown that simultaneously inhibiting efferocytosis by blocking TAMs and inhibiting indoleamine-2,3-dioxegenase 1 led to decreased immunosuppressive phenotypes and induced tumor regression (34). INCB081776 inhibited phagocytosis of human monocytes, consistent with anti-phagocytic properties of other MERTK inhibitors (35). In addition, INCB081776 effectively inhibited pMERTK upon activation of primary macrophages. Functionally, INCB081776 suppressed macrophage-mediated inhibition of T-cell proliferation and induced IFN-γ production, suggesting that MERTK inhibition plays a key role in this process. Therefore, inhibiting MERTK may play a role in reversing tumor-associated macrophages to an M1-like phenotype.

*In vivo*, INCB081776 possessed single-agent activity in the MBT-2, MC38, and 4T1 models, and antitumor activity was enhanced in combination with a PD-L1–blocking antibody in the MC38 and 4T1 models. Consistent with *in vitro* findings, combination treatment *in vivo* led to an increase in CD4^+^ and CD8^+^ T-cell proliferation, which was associated with an increase in IFN-γ production. Importantly, antitumor efficacy was lost when INCB081776 was dosed in immunodeficient animals bearing MBT-2 and 4T1 tumors, demonstrating that a functional immune system is required for efficacy in these syngeneic models. Overall, these data demonstrate a clear role of INCB081776 in modulating the immune system to promote antitumor responses.

Despite possessing antitumor activity that was dependent on the immune system, INB081776 also had direct antitumor activity in immunodeficient mice bearing sarcoma PDX tumors. A comparison between the nonresponsive CTG-1339 and responsive CTG-2041 sarcoma models demonstrated that while INCB081776 inhibited pAXL and pMERTK in both models, only pAKT was inhibited in CTG-2041. Our results are consistent with a prior report demonstrating that inhibition of pAKT is a biomarker of MERTK inhibition, although it is likely that inhibition of both pAXL and pMERTK contributed to the reduction of pAKT in the CTG-2041 model (36). These data indicate that AXL and MERTK are strong drivers of tumor growth in CTG-2041, and suggest that in the CTG-1339 model, there are other activated pathways that lead to AKT activation that are independent of AXL and MERTK. INCB081776 also modestly increased the levels of total MERTK. Although the reasons for this are not completely known, we speculate that INCB081776 may partially stabilize MERTK at the plasma membrane. The lack of response in other sarcoma PDX models tested may be due to different dependencies of these other tumors on other oncogenes and oncogenic pathways.

In summary, these data demonstrate that AXL/MERTK inhibition with INCB081776 hinders tumor growth by two distinct mechanisms—reversal of immunosuppression and ablation of intrinsic tumor cell survival signaling. We believe the profile of INCB081776 as a selective inhibitor of AXL and MERTK will provide an attractive potential treatment option for patients with cancer not only as a single agent, but also in rational combination with immunotherapeutic agents. A phase 1 clinical trial of INCB081776 is currently underway for patients with advanced solid tumors both as a single agent and in combination with PD-1 antibody INCMGA00012 (ClinicalTrials.gov identifier NCT03522142).

## Data Availability Statement

The original contributions presented in the study are included in the article/**Supplementary Material**, further inquiries can be directed to the corresponding author.

## Ethics Statement

The animal study was reviewed and approved by Incyte’s Animal Care and Use Committee.

## Author Contributions

JR-D, RW, YL, JH, SSe, XWa, Y-LL, and HK designed experiments; MF, KLa, PF, GY, CS, XWe, SSe, KK, KLi, MY, and CH conducted experiments; JR-D drafted the manuscript and WY, SSp, MC, PS, and HK edited the manuscript; all authors reviewed and approved the manuscript for submission.

## Funding

This study was funded by Incyte Corporation. The funder had the following involvement with the study: providing funding for laboratory materials to conduct these studies and salary support for authors.

## Conflict of Interest

JR-D, MF, KLa, PF, YL, GY, CS, XWa, SSe, KK, JH, MY, SSp, XWe, CH, WY, MC, and HK are employees of and own stock in Incyte Corporation. KLi, RW, Y-LL, and PS are former employees of and may own stock in Incyte Corporation.

## Abbreviations

BID, twice a day; CFSE, carboxyfluorescein succinimidyl ester; DMSO, dimethyl sulfoxide; FBS, fetal bovine serum; GAS6, growth arrest specific 6; IC50, half maximal inhibitory concentration; IFN, interferon; PBMC, peripheral blood mononuclear cell; PBS, phosphate-buffered saline; PD-L1, programmed death ligand; PDX, patient-derived xenograft; SOCS, suppressor of cytokine signaling; TAM, TYRO3, AXL, and MERTK; Teff, effector T cell.
