# Supplementary material for: A Potent and Selective Dual Inhibitor of AXL and MERTK Possesses Both Immunomodulatory and Tumor-Targeted Activity
Source: Front Oncol. 2020 Dec 7;10:598477. doi: 10.3389/fonc.2020.598477 (PMC7793849; doi:10.3389/fonc.2020.598477)

**A Potent and Selective Dual Inhibitor of AXL and MERTK Possesses Both Immunomodulatory and Tumor-Targeted Activity**

**Jonathan Rios-Doria*, Margaret Favata, Kerri Lasky, Patricia Feldman, Yvonne Lo, Gengjie Yang, Christina Stevens, Xiaoming Wen, Sarita Sehra, Kamna Katiyar, Ke Liu, Richard Wynn, Jennifer J. Harris, Min Ye, Susan Spitz, Xiaozhao Wang, Chunhong He, Yun-Long Li, Wenqing Yao, Maryanne Covington, Peggy Scherle, Holly Koblish**

Incyte Research Institute, Wilmington, DE, USA

***Supplementary Material***

**1 Supplementary Methods**

**1.1 Biochemical enzyme assays**

Phospho-AXL (pAXL), cMERTK, and Tyro3 kinase activities were measured by time-resolved fluorescence energy transfer assays. Autophosphorylation of AXL was carried out before the kinase assay by incubating the recombinant AXL protein (ThermoFisher Scientific; #PV4275) in buffer containing 50 mM Tris, pH 7.5, 0.2 mg/mL AXL, 5 mM ATP, 20 mM MgCl_2_, and 2 mM dithiothreitol (DTT) at room temperature for 1 hour. The kinase assay buffer contained 50 mM HEPES, pH 7.5, 10 mM MgCl_2_, 1 mM EGTA, 0.01% NP‑40, and 2 mM DTT. Enzyme solutions of 0.69 nM pAXL, or 0.088 nM cMERTK (Carna Biosciences, Kobe, Japan; #08-108) or 0.137 nM TYRO3 (Life Technologies, Carlsbad, CA, USA; PR7480A) were prepared in assay buffer. A 1-mM stock solution of peptide substrate Biotin-EQEDEPEGDYFEWLE-amide (Quality Controlled Biochemicals, Hopkinton, MA, USA) dissolved in dimethyl sulfoxide (DMSO) was diluted to 1 µM in assay buffer containing 2000 µM ATP. INCB081776 (15 nL) was dissolved in DMSO and transferred from compound plates to low-volume white 384-well assay plates (ProxiPlate, Perkin Elmer, Waltham, MA, USA). Different lots of INCB081776 used in biochemical assays were >98 pure. Enzyme solution (6 µL; or assay buffer for the enzyme blank) was added to the appropriate wells in each plate and incubated for 30 minutes. Then, 6 μL/well substrate solution was added to initiate the reaction. The plate was protected from light and incubated at room temperature (21°C) for 60 minutes (cMERTK and TYRO3) or 90 minutes (AXL). The reaction was stopped by adding 6-μL detection solution containing 50 mM Tris-HCl, pH 7.8, 150 mM NaCl, 0.05% BSA, 45 mM EDTA, 180 nM SA-APC (Perkin Elmer; CR130-100), and 3 nM Eu-W1024 anti-phosphotyrosine PY20 (Perkin Elmer; AD0067). The plate was incubated for 30 minutes at room temperature, and homogenous time-resolved fluorescence (HTRF) signal was measured on a PheraStar FS plate reader (BMG Labtech, Ortenberg, Germany). Percent inhibition was calculated for each concentration, and half maximal inhibitory concentration (IC_50_) value was generated from curve fitting with GraphPad Prism software (San Diego, CA, USA).

**1.2 ATP competition assay**

Compound reversibility was determined by measuring the recovery of cMERTK enzymatic activity after a rapid and large dilution of the cMERTK-inhibitor complex. cMERTK, ATP, and biotin-labeled peptide substrate were diluted in kinase assay buffer containing 50 mM HEPES, pH 7.5, 10 mM MgCl2, 1 mM EGTA, 0.01% NP-40, and 2 mM DTT. Detection reagents (240 nM SA-APC [Perkin Elmer; CR130-100] and 4 nM Eu-W1024 anti-phosphotyrosine PY20 [Perkin Elmer; AD0067]) were prepared in detection buffer containing 50 mM Tris-HCl, pH 7.8, 150 mM NaCl, 0.05% BSA, and 45 mM EDTA. To identify the inhibition mode of compounds, IC_50_ values were measured at different ATP concentrations (25, 100, 300, and 1000 μM final concentrations in reaction). INCB081776 was incubated with ATP and enzyme (66 pM cMERTK) in 8-µL assay buffer for an extended time (2 hours). Under these conditions, equilibrium among compounds, ATP, and enzyme was reached before reaction was started by addition of 4 μL of 1.5-μM biotin-labeled peptide. After 1 hour of incubation, the reaction was stopped by 4-µL detection reagent containing 60 mM EDTA, 240 nM SA-APC (Perkin Elmer; CR130-100), and 4 nM Eu-W1024 anti-phosphotyrosine PY20 (Perkin Elmer; #AD0067). Assay plates were read in HTRF mode by PheraStar plate reader after 30 minutes of incubation. Dose-response curves were fitted and IC_50_ values were plotted as a function of ATP concentrations.

**1.3 Kinase profiling**

Kinase profiling of INCB081776 was performed by Eurofins CEREP (Celle-Lévescault, France). The activity of INCB081776 at 200-nM concentration was evaluated against indicated kinases. Compound enzyme inhibition was calculated as a percent inhibition of control enzyme activity. Results showing an inhibition or stimulation higher than 50% are considered to represent significant effects of the test compounds.

**1.4 Characterization of Ba/F3 clones**

Ba/F3 cells were transfected with AXL, MERTK, or TYRO3 each containing an HA tag and a puromycin resistance cassette. Cell pools were diluted to 0.2-0.3 cells/well in medium lacking IL-3 and contained puromycin. Cells growing under these conditions were expanded and characterized by western blot for expression of AXL, MER, TYRO3 by Western blot using HA antibody (Sigma #H908). Selected clones with highest expression were used for the cell-based screens.

**1.5 AXL and MERTK expression on healthy donor PBMCs**

PBMCs were isolated from peripheral blood of healthy donors using Lymphoprep and Sepmate-50 tubes (STEMCELL Technologies, Vancouver, BC, Canada). The cells were counted and cell concentration adjusted to 2x10^6^/ mL in PBS. Cells were then stained with live dead aqua (ThernoFisher Scientific, MA, USA) followed by Fc block and surface staining. For surface staining, the cells were divided into four tubes for staining with PE anti-human AXL, PE IgG Isotype control for AXL, PE anti-human MERTK, and PE Isotype control for MERTK. The cells were labeled for antibodies to BV605 conjugated anti-CD42b (BD Biosciences), BV786 conjugated anti-CD16 (BD Biosciences), PerCP-Cy5.5 conjugated anti-CD14 (Invitrogen), and either PE anti-human MERTK (Biolegend San Diego, USA)/ PE Mouse IgG1, κ Isotype Ctrl Antibody (Biolegend)/ PE AXL Rabbit antibody (Cell Signaling Technology, MA, USA)/ PE Rabbit IgG Isotype Control (Cell Signaling Technology) for 30 min at 4^o^C. Cells were washed followed by acquisition on BD LSRFortessa. After gating for forward and side scatter, live cells were excluded for platelet aggregates (CD42b^+^ cells) as they are known to express AXL and MERTK. Expression of AXL and MERTK MFI was determined on the intermediate and classical populations of monocytes (CD14^++^CD16^+^ and CD14^++^ CD16^-^).

**1.6 Inhibition of pAXL in H1299 tumors**

H1299 tumor cells (CRL-5803, ATCC, Manassas, VA) were maintained in RPMI-1640 supplemented with 10% FBS. Five million H1299 cells were inoculated subcutaneously into the right hind flank of 6-8 week old female SCID mice (Charles River). A single dose of INCB081776 was orally administered at 3, 10, or 30 mg/kg in groups of 2-3 animals when tumors were approximately 300 mm^3^. Tumor samples were collected at 1, 2, 4, 8, or 16 hours post-dose administration. Tumor samples were collected and placed in tumor homogenate tubes (116913100, MP Biomedicals, Santa Ana, CA) and placed on dry ice until processing. Western Blotting for pAXL and AXL was performed as in the Materials and Methods.

**2 Supplementary Data**

**Supplementary Table 1.** Kinase profiling of INCB081776.

| **Kinase** | **Replicate 1**  **(% Inhibition of Control)** | **Replicate 2 (% Inhibition of Control)** | **Mean**  **(% Inhibition of Control)** | **Standard deviation** |
| --- | --- | --- | --- | --- |
| ALK | −0.9 | −3.4 | −2.2 | 1.8 |
| Akt1/PKB alpha | 8.0 | 4.8 | 6.4 | 2.3 |
| Akt2/PKB beta | 0.9 | −4.1 | −1.6 | 3.5 |
| Akt3/PKB gamma | −4.3 | −1.3 | −2.8 | 2.1 |
| ALK4 | −30.0 | −24.7 | −27.4 | 3.7 |
| AMPK alpha | 5.6 | 3.4 | 4.5 | 1.6 |
| Arg kinase | −5.2 | −4.4 | −4.8 | 0.6 |
| AurA/Aur2 kinase | 7.7 | 11.5 | 9.6 | 2.7 |
| Axl kinase | 94.8 | 94.4 | 94.6 | 0.3 |
| BMPR1A | 4.8 | 7.3 | 6.1 | 1.8 |
| Brk | −5.0 | 3.8 | −0.6 | 6.2 |
| BRSK1 | −3.1 | −1.1 | −2.1 | 1.4 |
| CaMK1 alpha | −1.6 | 4.6 | 1.5 | 4.4 |
| CaMK1 delta | 0.8 | −2.3 | −0.8 | 2.2 |
| CaMK2 alpha | 0.4 | 3.2 | 1.8 | 2.0 |
| CDC2/CDK1 | 2.4 | 10.1 | 6.3 | 5.4 |
| CDK2 | −0.4 | 3.6 | 1.6 | 2.8 |
| CDK3 | 2.4 | −0.3 | 1.1 | 1.9 |
| CDK4 | −0.5 | 0.9 | 0.2 | 1.0 |
| CDK6 | −9.5 | 1.7 | −3.9 | 7.9 |
| CDK7 | 0.8 | 2.0 | 1.4 | 0.8 |
| CDK8 | −0.6 | 6.2 | 2.8 | 4.8 |
| CDK9 | 6.5 | −1.9 | 2.3 | 5.9 |
| CHK1 | −1.7 | −0.6 | −1.2 | 0.8 |
| CHK2 | 8.6 | 6 | 7.3 | 1.8 |
| CK2 | 11.2 | −0.2 | 5.5 | 8.1 |
| c-kit | −9.5 | −7.7 | −8.6 | 1.3 |
| CLK1 | 1.5 | −0.8 | 0.4 | 1.6 |
| c-Met | 62.0 | 43 | 52.5 | 13.4 |
| COT kinase | −13.5 | −12.9 | −13.2 | 0.4 |
| CRIK | −11.5 | 4.8 | −3.4 | 11.5 |
| DAPK2 | 5.5 | −2.3 | 1.6 | 5.5 |
| DCAMKL1 | 4.2 | 5.9 | 5.1 | 1.2 |
| DCAMKL2 | 3.2 | 4.4 | 3.8 | 0.8 |
| DDR2 | −9.7 | −16.6 | −13.2 | 4.9 |
| DLK1 | −4.3 | −1.3 | −2.8 | 2.1 |
| DRAK1 | 7.7 | 9.3 | 8.5 | 1.1 |
| DYRK1a | 17.7 | 1.4 | 9.6 | 11.5 |
| DYRK2 | 4.9 | 7.1 | 6.0 | 1.6 |
| EGFR | −6.6 | −12.3 | −9.5 | 4.0 |
| EphA1 | −2.2 | −19 | −10.6 | 11.9 |
| EphA2 | −17.7 | 5.4 | −6.2 | 16.3 |
| EphA3 | −10.5 | 0.6 | −5.0 | 7.8 |
| EphA4 | 2.6 | 1.1 | 1.9 | 1.1 |
| EphA5 | 6.0 | 1.4 | 3.7 | 3.3 |
| EphA7 | 1.5 | −1.4 | 0.1 | 2.1 |
| EphB1 | −24.1 | −5.7 | −14.9 | 13.0 |
| EphB2 | 1.3 | −5.8 | −2.3 | 5.0 |
| EphB4 | 11.4 | 7.0 | 9.2 | 3.1 |
| ERK1 | 0.9 | −4.1 | −1.6 | 3.5 |
| ERK2 | 5.6 | 1.2 | 3.4 | 3.1 |
| FAK | −1.3 | −6.6 | −4.0 | 3.7 |
| Fes kinase | 5.9 | 1.5 | 3.7 | 3.1 |
| Fer kinase | −3.3 | −4.5 | −3.9 | 0.8 |
| FGFR1 | −8.4 | −4.7 | −6.6 | 2.6 |
| FGFR2 | −4.3 | −8.0 | −6.2 | 2.6 |
| FGFR3 | 0.2 | −2.4 | −1.1 | 1.8 |
| FGFR4 | −1.5 | −6.6 | −4.1 | 3.6 |
| Fgr kinase | 21.8 | 14.3 | 18.1 | 5.3 |
| FLT-1 kinase | −6.5 | 1.6 | −2.5 | 5.7 |
| FLT-3 | 8.7 | 32.6 | 20.7 | 16.9 |
| FLT-4 | −11.1 | −8.8 | −10.0 | 1.6 |
| Fms/CSFR | 1.0 | 11.9 | 6.5 | 7.7 |
| FRK | 3.3 | 11.4 | 7.4 | 5.7 |
| Fyn kinase | −0.3 | 5.7 | 2.7 | 4.2 |
| GCK | 10.0 | −2 | 4.0 | 8.5 |
| GRK2 | 6.0 | 0.1 | 3.1 | 4.2 |
| GRK3/BARK2 | −0.2 | 0 | −0.1 | 0.1 |
| GSK3 alpha | −4.1 | −2.9 | −3.5 | 0.8 |
| GSK3 beta | −5.0 | 6.1 | 0.6 | 7.8 |
| HER2/ErbB2 | −1.7 | −2.7 | −2.2 | 0.7 |
| HGK | −11.2 | 9.4 | −0.9 | 14.6 |
| HIPK2 | −5.2 | 0.9 | −2.2 | 4.3 |
| IGF1R | 6.9 | 3.4 | 5.2 | 2.5 |
| IKK alpha | −4.3 | −4 | −4.2 | 0.2 |
| IKK beta | 3.4 | −0.9 | 1.3 | 3.0 |
| IKK epsilon | 0.0 | −4.2 | −2.1 | 3.0 |
| IRAK1 | −1.8 | 4.1 | 1.2 | 4.2 |
| IRAK4 | 18.5 | 21.6 | 20.1 | 2.2 |
| IRK | 0.5 | 1 | 0.8 | 0.4 |
| IRR kinase | 24.0 | 41.8 | 32.9 | 12.6 |
| ITK | 7.8 | 6.9 | 7.4 | 0.6 |
| JAK1 | 2.2 | 1.6 | 1.9 | 0.4 |
| JAK2 | −15.3 | −10.9 | −13.1 | 3.1 |
| JAK3 | 9.7 | 4.6 | 7.2 | 3.6 |
| JNK1 | −2.6 | −4.5 | −3.6 | 1.3 |
| JNK2 | 7.2 | 10.8 | 9.0 | 2.5 |
| JNK3 | −4.7 | −2.1 | −3.4 | 1.8 |
| KDR kinase | −1.8 | 2.1 | 0.2 | 2.8 |
| Lck | 1.6 | −3.6 | −1.0 | 3.7 |
| LIMK1 | 2.6 | 4.8 | 3.7 | 1.6 |
| LTK | −6.9 | −1.3 | −4.1 | 4.0 |
| Lyn A | 15.3 | 39.1 | 27.2 | 16.8 |
| Lyn B | −5.1 | −4.4 | −4.8 | 0.5 |
| MEK5 | −0.1 | −9.9 | −5.0 | 6.9 |
| MEKK4 | −3.7 | −4.9 | −4.3 | 0.8 |
| MAPKAPK2 | 5.1 | −3.6 | 0.8 | 6.2 |
| MAPKAPK5 | −7.1 | 11.9 | 2.4 | 13.4 |
| MARK1 | 6.7 | −0.8 | 3.0 | 5.3 |
| MARK3 | 15.5 | 10.6 | 13.1 | 3.5 |
| MEKK3 | 1.0 | 0.3 | 0.7 | 0.5 |
| NIM1 kinase | 1.6 | −2.5 | −0.5 | 2.9 |
| MLK1 | −0.7 | 3.2 | 1.3 | 2.8 |
| MNK1 | −3.1 | 2.6 | −0.3 | 4.0 |
| MLK2 | 22.9 | 18.2 | 20.6 | 3.3 |
| MSK2 | 6.7 | 7.1 | 6.9 | 0.3 |
| MST1 | 11.8 | 11.4 | 11.6 | 0.3 |
| MST3 | −10.0 | −1 | −5.5 | 6.4 |
| MusK | −1.7 | 1 | −0.4 | 1.9 |
| MYT1 kinase | 10.7 | 6.1 | 8.4 | 3.3 |
| NDR1 kinase | −0.4 | 2.4 | 1.0 | 2.0 |
| NEK2 | −6.7 | 2.6 | −2.1 | 6.6 |
| NEK4 | 1.9 | −0.3 | 0.8 | 1.6 |
| NEK6 | −1.5 | −29.7 | −15.6 | 19.9 |
| NIK | −1.3 | −14.9 | −8.1 | 9.6 |
| NuaK1 | 3.1 | 5 | 4.1 | 1.3 |
| P38 alpha | −12.2 | −18.9 | −15.6 | 4.7 |
| P38 delta | −4.3 | −3.2 | −3.8 | 0.8 |
| p70S6K | −0.8 | 4.1 | 1.7 | 3.5 |
| p70S6K beta | 6.0 | 7.8 | 6.9 | 1.3 |
| PAK2 | 0.2 | −10.2 | −5.0 | 7.4 |
| PAK4 | 6.8 | −1.1 | 2.9 | 5.6 |
| PCTAIRE kinase | −1.2 | 1.7 | 0.3 | 2.1 |
| PDGFR alpha | 5.6 | 4.4 | 5.0 | 0.8 |
| PDGFR beta | −1.5 | −0.2 | −0.9 | 0.9 |
| PDK1 | 9.5 | 2.8 | 6.2 | 4.7 |
| PEK | 18.0 | 20.2 | 19.1 | 1.6 |
| PhK gamma 1 | −2.7 | 4.1 | 0.7 | 4.8 |
| PhK gamma 2 | −0.4 | −11 | −5.7 | 7.5 |
| Pim1 kinase | 7.8 | 11.1 | 9.5 | 2.3 |
| Pim2 kinase | −8.2 | 3.4 | −2.4 | 8.2 |
| PKA | 3.3 | 8.8 | 6.1 | 3.9 |
| PKC alpha | −7.7 | −1.2 | −4.5 | 4.6 |
| PKC Beta 2 | −2.6 | 0 | −1.3 | 1.8 |
| PKG1 beta | 7.5 | 10 | 8.8 | 1.8 |
| PKN1 | −3.9 | −10.2 | −7.1 | 4.5 |
| PKN2 | −13.6 | −10.4 | −12.0 | 2.3 |
| PLK1 | 0.4 | 1.4 | 0.9 | 0.7 |
| PLK2 | 4.9 | 2.4 | 3.7 | 1.8 |
| PRKX | −10.7 | −5.6 | −8.2 | 3.6 |
| PYK2 | −0.8 | −4.6 | −2.7 | 2.7 |
| B-raf | −2.5 | −8.8 | −5.7 | 4.5 |
| RAF-1 kinase | −8.7 | 15.5 | 3.4 | 17.1 |
| Ret | 4.9 | 3.6 | 4.3 | 0.9 |
| RIPK2 | −3.6 | −5.3 | −4.5 | 1.2 |
| ROCK1 | −12.1 | −2.7 | −7.4 | 6.6 |
| ROCK2 | 4.3 | −5.3 | −0.5 | 6.8 |
| Ron | −9.0 | 1.3 | −3.9 | 7.3 |
| RSK1 | 3.5 | 2.9 | 3.2 | 0.4 |
| RSK2 | −0.4 | −3.3 | −1.9 | 2.1 |
| SGK1 | −8.8 | −1.4 | −5.1 | 5.2 |
| SGK3 | 0.9 | 4.3 | 2.6 | 2.4 |
| SIK | 1.2 | −4.2 | −1.5 | 3.8 |
| STK33 | 6.7 | 2.8 | 4.8 | 2.8 |
| Syk | −4.4 | −10.4 | −7.4 | 4.2 |
| TAK1-TAB1 | −11.6 | −18.4 | −15.0 | 4.8 |
| TAOK2 | 30.4 | 17.9 | 24.2 | 8.8 |
| TBK1 | 8.2 | 16.8 | 12.5 | 6.1 |
| Tnk1 | −2.6 | 5.4 | 1.4 | 5.7 |
| TRKA | −2.4 | −12.9 | −7.7 | 7.4 |
| TRKB | −2.3 | 19.4 | 8.6 | 15.3 |
| TRKC | 11.7 | −5.3 | 3.2 | 12.0 |
| TSSK1 | 3.0 | 3.4 | 3.2 | 0.3 |
| TTK | −2.8 | 2.7 | 0.0 | 3.9 |
| TXK | 4.6 | −8.3 | −1.9 | 9.1 |
| Tyk2 | 7.5 | −14 | −3.3 | 15.2 |
| Tyro3 | 20.6 | 4.5 | 12.6 | 11.4 |
| ULK1 | −4.9 | 11.1 | 3.1 | 11.3 |
| Wee1 | 13.1 | −5.4 | 3.9 | 13.1 |
| WNK2 | −4.2 | 4.7 | 0.3 | 6.3 |
| WNK3 | 1.7 | 1.9 | 1.8 | 0.1 |
| WNK4 | 1.1 | 11.2 | 6.2 | 7.1 |
| Yes kinase | 2.4 | 2.4 | 2.4 | 0.0 |
| ZAP70 | −5.7 | −5.3 | −5.5 | 0.3 |

**Supplementary Table 2.** Tumor Growth Inhibition of INCB081776 in sarcoma PDX models.

| **Model** | **Subtype** | **TGI (%)** | **P value** |
| --- | --- | --- | --- |
| CTG-0898 | Liposarcoma | -1 | 0.99 |
| CTG-1182 | Leiomysarcoma | -6 | 0.89 |
| CTG-1302 | Leiomysarcoma | 48 | 0.001 |
| CTG-1339 | Osteosarcoma | 5 | 0.91 |
| CTG-2041 | Angiosarcoma | 94 | <0.0001 |
| CTG-1094 | UPS | 23 | 0.27 |
| CTG-0709 | Pleomorphic spindle | 8 | 0.99 |
| CTG-2426 | Myxofibrosarcoma | 42 | 0.03 |
| CTG-2265 | UPS | 13 | 0.94 |
| CTG-0663 | Fibrous histiocytoma | -27 | 0.65 |
| CTG-1861 | GIST | 74 | <0.0001 |
| CTG-0980 | Angiosarcoma | 19 | 0.83 |
| CTG-2250 | Angiosarcoma | -9 | 0.99 |
| CTG-1572 | Angiosarcoma | 2 | 0.99 |

**Supplementary Figure 1.** INCB081776 has an ATP-competitive mode of inhibition. INCB081776 was incubated with indicated concentrations of ATP and cMERTK enzyme for 2 hours. Dose-response curves were fitted and half maximal inhibitory concentration (IC_50_) values were plotted as a function of ATP concentrations. SD = standard deviation.

**
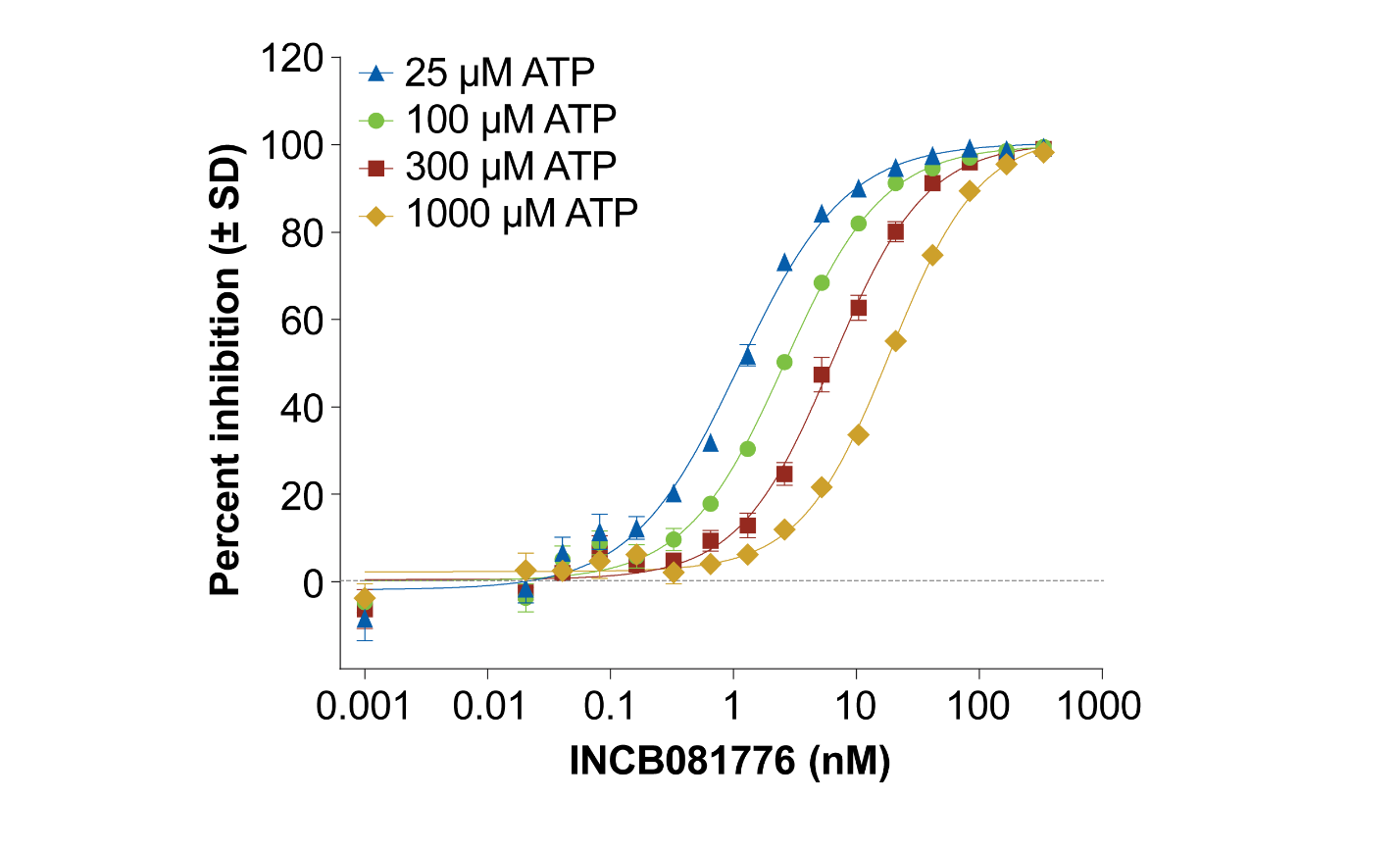
**

**Supplementary Figure 2.** FACS gating strategy for phagocytosis assay. Human whole blood was treated with DMSO Control and peripheral blood mononuclear cells (PBMCs) were isolated and labeled with anti-CD14 and anti-CD16 followed by acquisition. (**A**) DMSO control prior to addition of YG fluorescent microspheres. (**B**) DMSO Control, 80 seconds after addition of fluorescent microspheres. The third and fourth columns are the histograms for YG+ population from CD14++CD16+ intermediate monocytes and CD14++CD16- classical monocytes, respectively. Data are representative of three healthy donors.


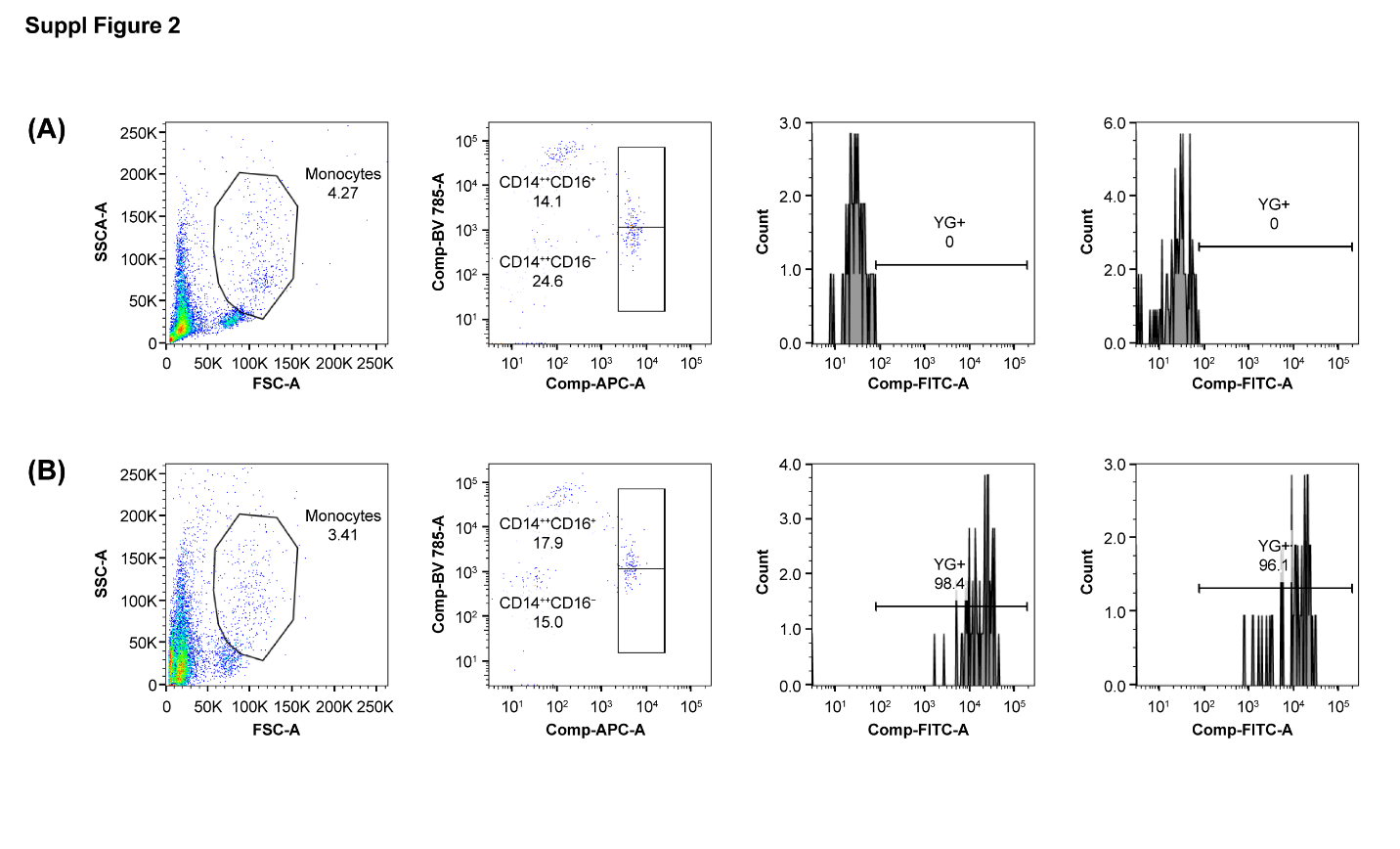


**Supplementary Figure 3.** Expression of AXL and MERTK on intermediate (CD14++CD16+) and classical (CD14++CD16-) monocytes from healthy donor PBMCs and phagocytosis assay in CD14++CD16- classical monocytes. Levels of AXL and MERTK expression were determined by staining with CD14, CD16, CD42b antibodies (as described in Materials and Methods) and PE-conjugated isotype control or PE conjugated AXL or MERTK antibodies prior to ﬂow cytometric analysis. (**A**). The MFI of MERTK and (**B**) AXL expression in the intermediate (CD14++CD16+) and classical (CD14++CD16-) monocyte subsets is shown. Data from two healthy donors are shown. (**C**), Inhibition of phagocytosis of CD14++CD16- classical monocytes by INCB081776. Human whole blood was treated with INCB081776 at 10 nM and 100 nM along with DMSO control. Fluorescent microspheres were added to peripheral blood mononuclear cells (PBMCs) prelabeled with anti-CD14 and anti-CD16, and acquisition performed at intervals over a course of 4 minutes. Plot indicating the mean of the uptake of fluorescent microspheres by the classical (CD14++CD16-) monocyte population over time is shown. Data are representative of three healthy donors.


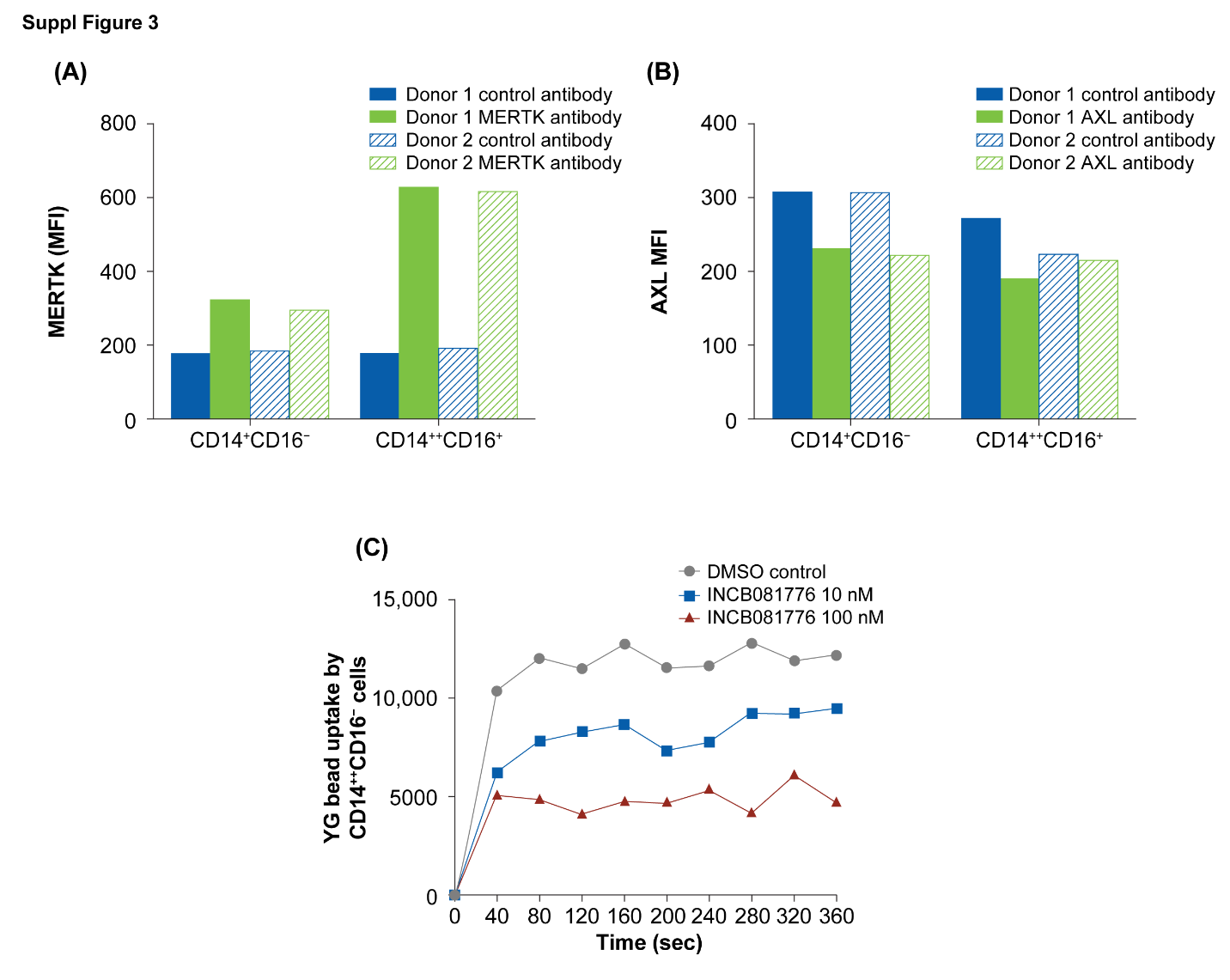


**Supplementary Figure 4.** Inhibition of pAXL in H1299 tumors. (**A**) H1299 tumor-bearing SCID mice (n=2-3 per group) were given a single dose of INCB081776 at 3, 10, or 30 mg/kg, or vehicle control (VEH). Tumors were collected 2 hours post-dose and analyzed for pAXL and total AXL levels. (**B**) H1299 tumor-bearing SCID mice were given a single dose of INCB081776 at 30 mg/kg. Tumors (n=3 per group) were collected at 1, 2, 4, 8 , or 16 hours post-dose and analyzed for pAXL and total AXL levels.

**
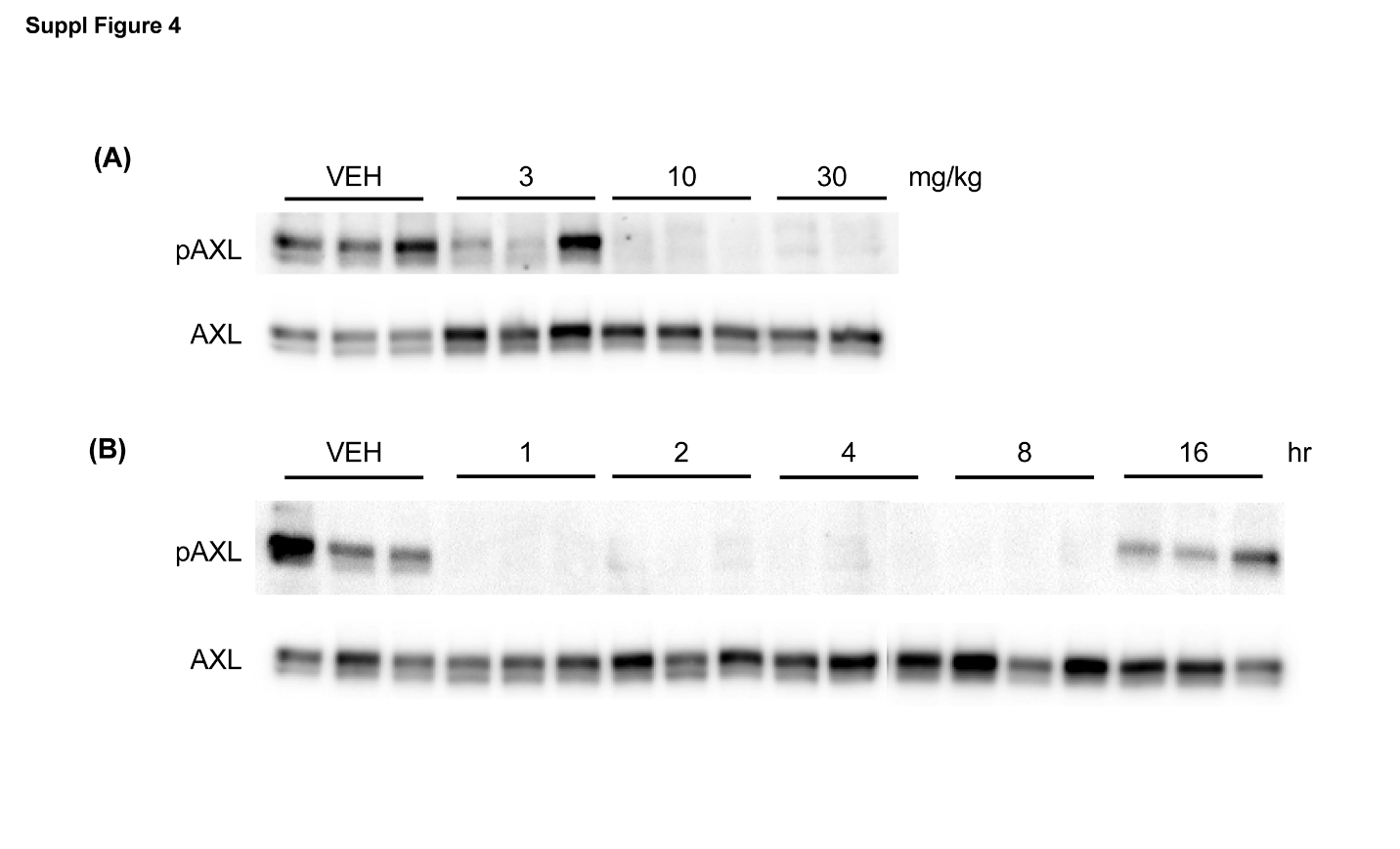
**

**Supplementary Figure 5.** No change in body weight in tumor-bearing mice treated with INCB0816776. (**A**) MBT-2 tumor-bearing or (**B**) 4T1 tumor-bearing mice were dosed with INCB081776 as shown in Figure 3A and 3B. *N* = 12 mice per group. SEM = standard error of the mean


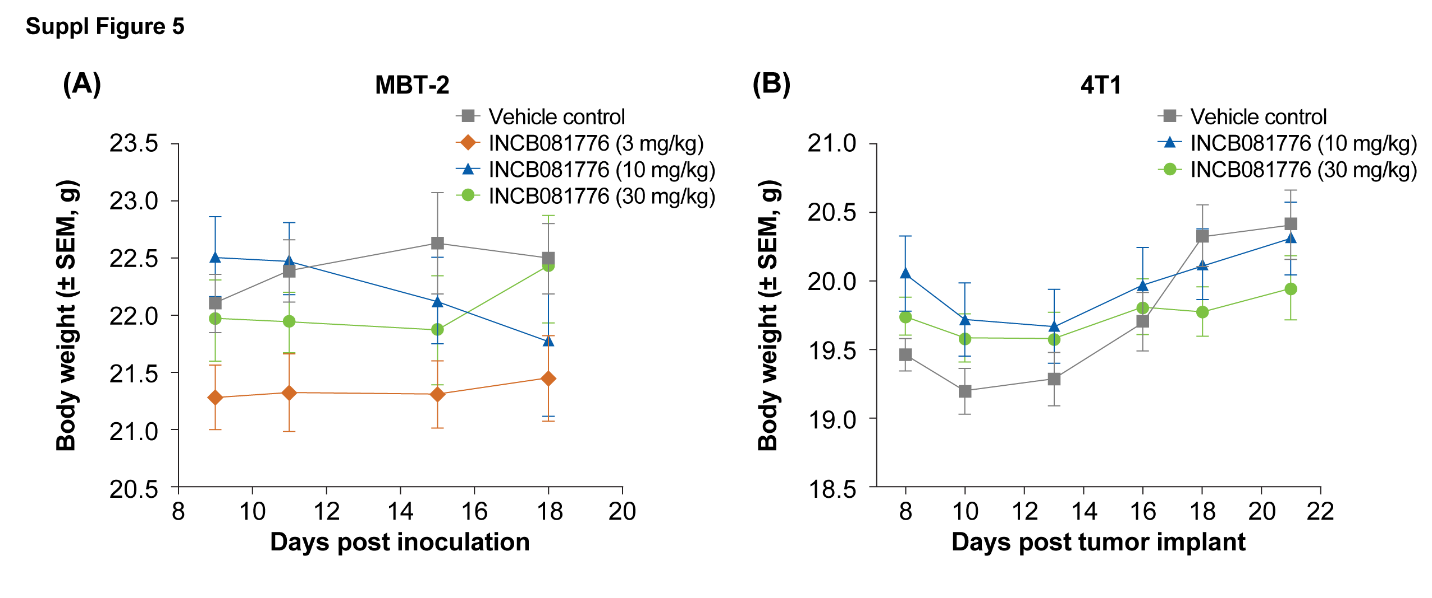


**Supplementary Figure 6.** INCB081776 is inactive in MBT-2 and 4T1 tumor–bearing nude mice. (**A**) Nude mice bearing established MBT-2 tumors were dosed with INCB081776 at 3, 10, and 30 mg/kg orally twice a day. *N* = 10 mice per group. (**B**) Nude mice bearing established 4T1 tumors were dosed with INCB081776 at 10 and 30 mg/kg orally twice a day. *N* = 10 mice per group. SEM = standard error of the mean.

**
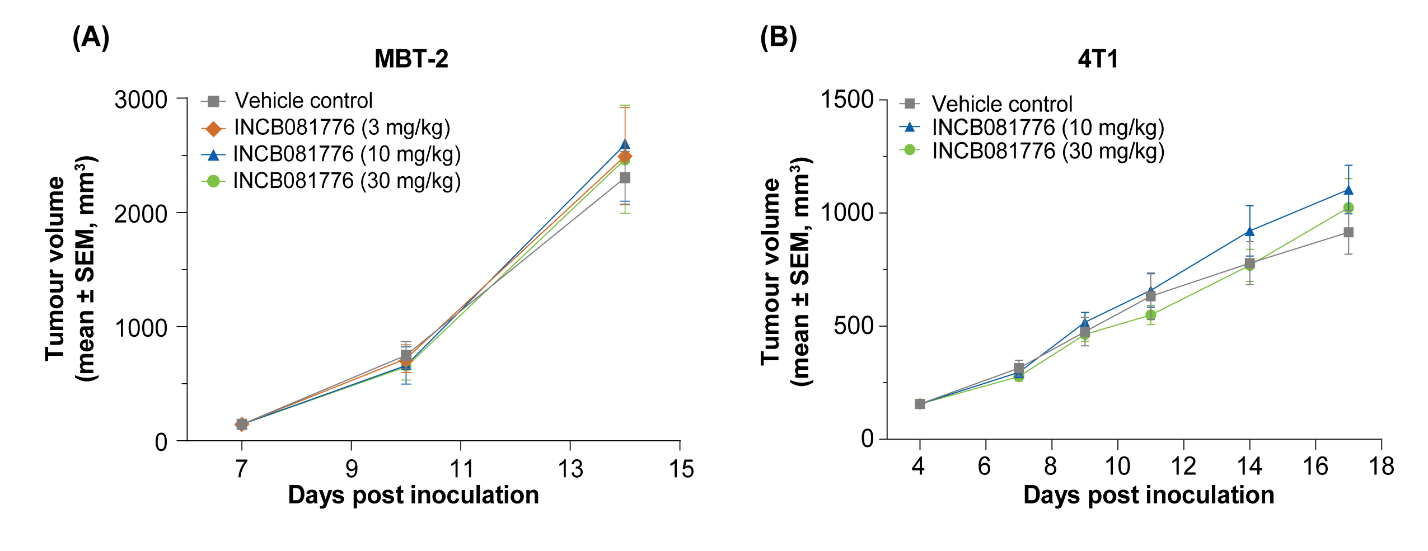
**

**Supplementary Figure 7.** MBT-2 tumor bearing C3H or nude mice were dosed with INCB081776 continuously in antitumor efficacy studies as previously described. On the last day of the studies in MBT-2 tumor-bearing C3H mice (day 18) or nude mice (day 14), plasma was collected at 1, 2, 4, 8, and 16 hours (C3H mice) or 2, 4, and 16 hours (nude mice) after the last oral administration of INCB081776. Plasma pharmacokinetics of INCB081776 in (**A**) MBT-2 tumor bearing C3H mice or (**B**) nude mice are shown.

**
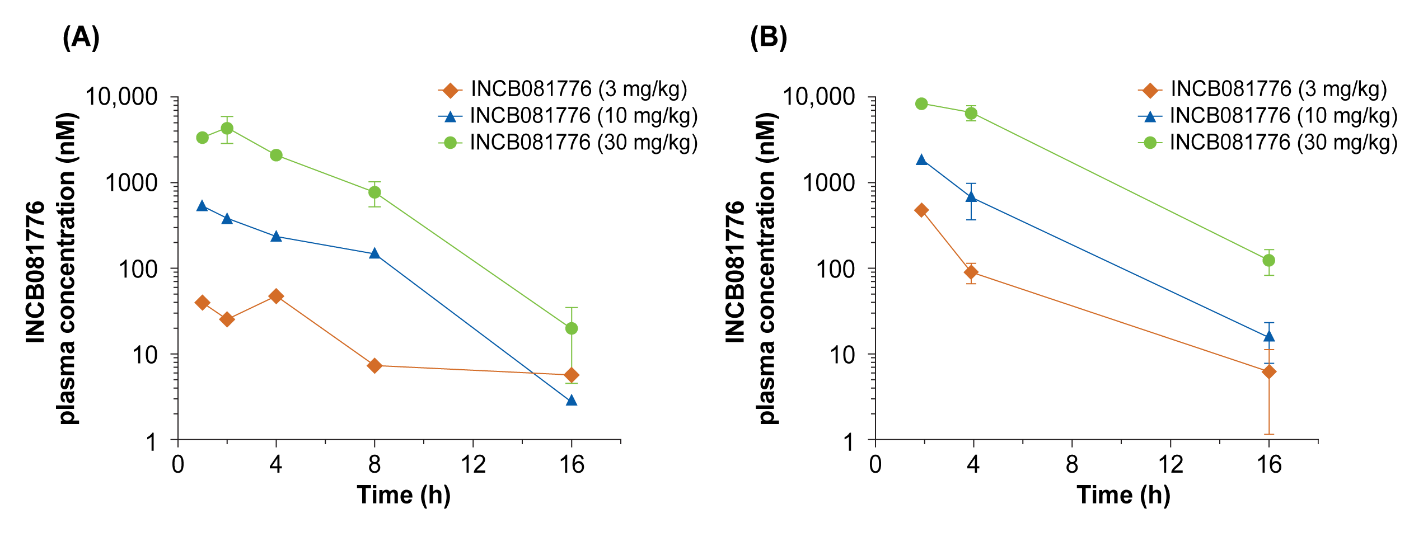
**

**Supplementary Figure 8.** INCB081776 displays enhanced antitumor activity in combination with anti–programmed death ligand 1 (PD-L1) in the 4T1 model. BALB/c mice bearing established 4T1 tumors were administered INCB081776 orally twice a day at the indicated dose levels, anti–PD-L1 twice a week, or the combination. *N* = 12 mice per group. **p* < 0.05. SEM = standard error of the mean; TGI = tumor growth inhibition.


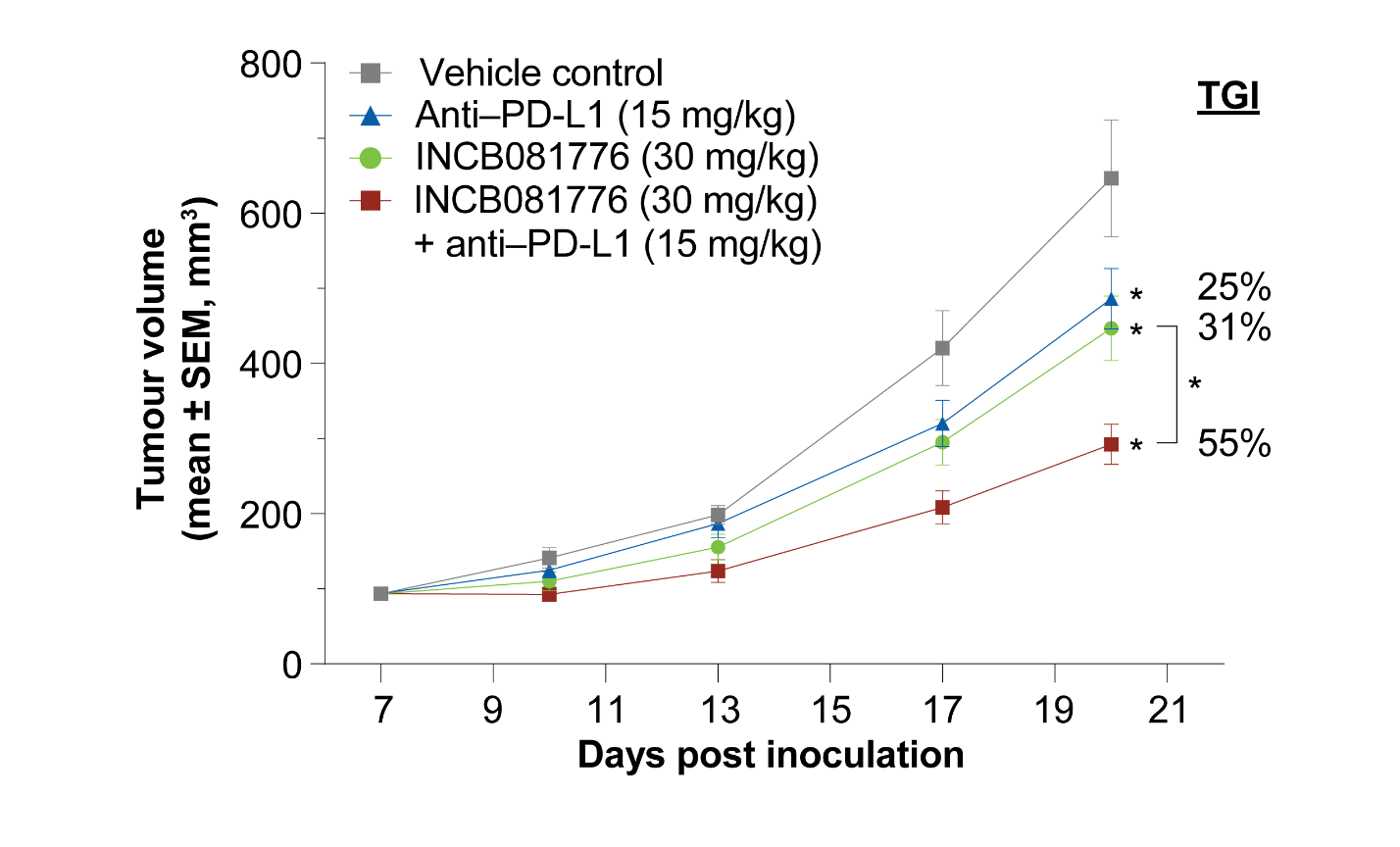

Supplement: Supplementary file 1 [file DataSheet_1.docx]
